# Assessing the importance of sex and disease-specific anatomy in electrophysiology and mechanical simulations with a newly developed public virtual cohort of four-chamber heart models

**DOI:** 10.1371/journal.pcbi.1014325

**Published:** 2026-06-02

**Authors:** José Alonso Solís-Lemus, Rosie K. Barrows, Cristobal Rodero, Marina Strocchi, Natalie Montarello, Nishant Lahoti, Cesare Corrado, Abdul Qayyum, Shahrokh Rahmani, Caroline Roney, Gernot Plank, Christoph Augustin, Hao Xu, Alistair Young, Pras Pathmanathan, Ronak Rajani, Steven A. Niederer

**Affiliations:** 1 National Heart and Lung Institute, Imperial College London, London, United Kingdom; 2 Cardiac Rhythm Management, Medtronic, London, United Kingdom; 3 School of Biomedical Engineering and Imaging Sciences, King’s College London, London, United Kingdom; 4 School of Engineering and Materials Science, Queen Mary University of London, London, United Kingdom; 5 Gottfried Schatz Research Center, Division of Medical Physics and Biophysics, Medical University of Graz, Graz, Austria; 6 BioTechMed-Graz, Graz, Austria; 7 Food and Drug Administration, Silver Spring, Maryland, United States of America; 8 Alan Turing Institute, London, United Kingdom; University of California San Diego, UNITED STATES OF AMERICA

## Abstract

This work presents a study on how differences in cardiac anatomy attributed to sex and disease can influence cardiac electrophysiology and mechanics using a virtual cohort of four-chamber heart models. Patient anatomy varies across sex and disease. However, capturing this variation in in-silico studies remains poorly accounted for, with studies often using either single representative cases or imbalanced virtual cohorts. Whole-heart electromechanics models incorporate the patient’s anatomy, electrophysiology and mechanics across different scales, from molecular, tissue and whole-heart and circulatory system levels. However, cardiac models are typically built from one or a small number of anatomies, with sex rarely reported and the effects of anatomical variability, which include those due to sex or disease, largely unexplored. This limits clinical translation and reduces regulatory credibility. We developed fifty patient-specific anatomical models of 25 male and 25 female hearts in heart failure and control cases. We ran benchmark passive inflation and paced activation simulations with consistent parameters and boundary conditions across cases to isolate the impact of anatomical variations with sex and disease. Heart failure models exhibited increased chamber volumes, larger volume changes during inflation, and delayed activation times relative to controls. These trends were consistent across sexes, although right ventricular activation showed a significant sex-based difference. Variations in anatomy with sex and disease have a significant impact on cardiac simulations, which support the inclusion of multiple heart anatomical models in in-silico trials. The resulting virtual cohort captures key anatomical variability and is publicly available, along with the underlying code (see Data Availability statement).

## Author summary

Understanding how sex and disease affect the heart’s electrical and mechanical behaviour is essential for developing accurate and credible computational models. In this study, we built a balanced virtual cohort of fifty patient-specific, four-chamber heart models from male and female individuals with and without heart failure. We designed a single, streamlined pipeline to generate these models and ran benchmark simulations of electrical activation and passive inflation to evaluate how anatomical differences influence cardiac function. We found that heart failure models showed larger chamber volumes, greater volume changes, and delayed activation times compared with controls. These patterns were consistent between sexes, although right ventricular activation showed a clear sex-related difference. Our results demonstrate that anatomical variability across sex and disease has a measurable effect on cardiac simulations. By releasing this virtual cohort and its underlying pipeline publicly, we aim to support reproducible and transparent in-silico studies and to advance the use of virtual populations in regulatory and clinical applications.

## 1. Introduction

Patient-specific computational models and simulations of the heart have increasingly been used to develop and guide clinical therapies [1], supporting the design [2,3], evaluation [4,5], and delivery [6,7] of innovative clinical therapies. These models could advance personalised medicine by enabling the development and in-silico testing of therapies tailored to the anatomical and physiological features of individual patients. However, the creation of credible and trustworthy cardiac models remains challenging [8,9].

There is growing support for the use of simulations for regulatory submission by the Medicines and Healthcare products Regulatory Agency [10], European Medicines Agency [11], and U.S. Food and Drug Administration (FDA) [9,12]. In particular, the FDA has identified scientific gaps and challenges that have limited the credibility of simulation models intended for medical use, including (i) insufficient analytic methods, (ii) lack of established credibility assessment tools, (iii) absence of best-practice guidelines, and (iv) limited availability of high-quality, comprehensive data sets [12].

Data scarcity remains a fundamental obstacle to the development of robust cardiac models, particularly the lack of high-quality, sex-balanced, disease-specific patient data. Several studies have attempted to bridge this gap through the creation of virtual cohorts that can be used for in-silico trials [13,14]. Despite these advances, addressing sex balance in cardiac modeling databases is a persisting challenge.

The cohort presented by Strocchi et al. [13] has one female patient, while the one presented by Rodero et al. [14] has 6 out of 20. Finally, in the study by Roney et al. [15] on predicting atrial fibrillation recurrence, 28 of the 99 patients were female. Large virtual heart cohorts have begun to emerge, enabling in-silico trials and population-level analyses [16–18]. For example, the Strocchi et al. [13] database has been widely used for studies on valve mechanics [19], fibrosis detection [20], and arrhythmia modeling [21].

While these cohorts represent valuable tools for the cardiac modelling community, they also highlight the challenge of having balanced representation of sex or disease subtypes, and workflows for generating new whole-heart cohorts remain poorly defined.

Sex bias is a complex, multi-factorial problem that affects the availability and generalisability of data used for computational models and simulations [22]. Simulation studies can take considerable time and computational resources, which makes it especially relevant to consider whether anatomical differences associated with sex or disease influence the results. The impact of such variation on simulation outcomes as well as on cardiac function is not frequently explored or understood. Understanding this relationship is key to determining when sex-specific anatomical data should be included in model development and evaluation.

Testing the importance of anatomical variability requires creating sex balanced virtual cohorts. However, building a model of the whole heart is a labour-intensive and technically demanding task. Available solutions involve multiple stages: image segmentation, mesh generation, and the assignment of fibre orientations. Some approaches have explored the use of deep learning to automate aspects of mesh generation [23]. However, these methods are typically limited to single cardiac chambers rather than full four-chamber models.

CemrgApp [24] is an open-source medical imaging platform with image processing toolkits for cardiovascular research, that has a low barrier to entry and low learning curve, putting reproducibility and best practices at the forefront. CemrgApp has enabled semi-automated creation of high-quality left atrial models and has been employed in studies involving atrial fibrosis characterisation [25], motion quantification [26], and regional strain analysis [27]. However, the extension of such tools to comprehensive whole-heart modelling, including all four chambers, has not been systematically demonstrated or standardised.

In this study, we developed CEMRG Heartbuilder, a Python-based library designed to systematically generate patient-specific, four-chamber heart models from clinical computerised tomography (CT) data. We applied the workflow to a new cohort of 50 patients (balanced by sex), classified into three clinical groups: controls (*n* = 26), heart failure with narrow QRS (*n* = 12), and heart failure with wide QRS (*n* = 12). For each case, models were created from a CT scan to a simulation-ready mesh with fibres, and subsequently used to run benchmark electrophysiological and mechanical simulations. The simulations demonstrated the feasibility and robustness of the approach, and, through the use of uniform material properties, isolated the impact of disease and sex differences in anatomy on reference mechanical and electro physiological simulations.

**Fig 1:**
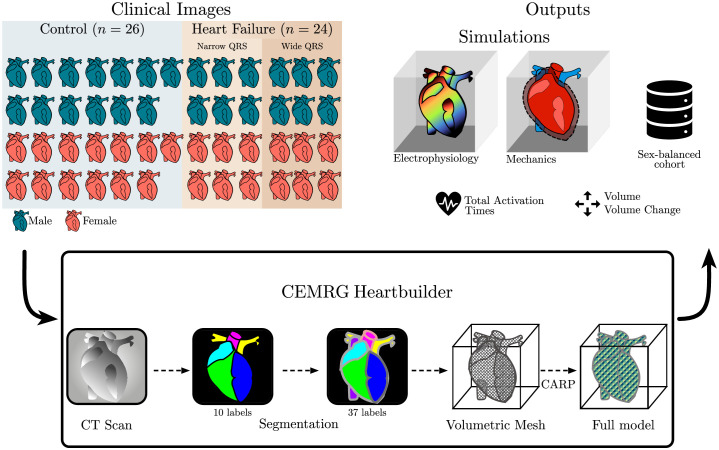
Cohort creation and pipeline overview. Starting from clinical CT scans, the workflow performs multi-label segmentation and mesh generation to create simulation-ready models with assigned fibre orientations. A new dataset of 50 patients (26 controls, 24 with heart failure subtypes: narrow QRS and wide QRS) balanced by sex is processed. Resulting models are used in simulations of cardiac electrophysiology and mechanics to extract clinically relevant measurements. Heart icons in this figure were derived from a CC0 image obtained from openclipart.org (https://openclipart.org/246884) and modified to fit the figure design.

Fig 1 provides an overview of the pipeline and the resulting cohort.

## 2. Study population

Fifty cases were processed, creating patient-specific four-chamber heart models. The cohort was split into three groups: Controls (*C*, *n* = 26), Heart Failure (*HF*) with narrow QRS (*HF*_*N*_, *n* = 12), and HF with wide QRS (*HF*_*W*_, *n* = 12); each group is balanced by sex (50% Female). A QRS duration of less than 120 ms is considered narrow. Each case consisted of a CT scan, acquired using a consistent imaging protocol. The reconstructed meshes represent the cardiac anatomy in a static reference configuration. Subsequent mechanical simulations apply prescribed endocardial pressures from this baseline geometric state, enabling comparison of anatomical effects across the cohort.

A summary of the cohort’s demographics is presented in Table 1. Summary statistics include the number of participants per group, age, ejection fraction (EF) as a percentage, and QRS duration in milliseconds (ms). Male control cases were patients with no known cardiac disease or symptoms. Five cases had no echocardiography data available and 1 case had no ECG.

**Table 1:** Summary of the 50-patient cohort demographics, grouped by sex and condition. See text for full description of the cohort and the conditions. Abbreviations: M = Male, F = Female, HF = Heart Failure, *HF*_*N*_=HF with Narrow QRS duration, *HF*_*W*_=HF with Wide QRS duration.

	Total	Age	EF (%)	QRS duration (ms)
*M*_*C*_	13	53.4 ± 12	58.7 ± 4(*n* = 8)	90.3 ± 8, (*n* = 12)
$M_{HF_{N}}$	6	53.8 ± 13.8	34.4 ± 14.2	103 ± 7.8
$M_{HF_{W}}$	6	67.3 ± 16	38.5 ± 8	163.8 ± 18
*F*_*C*_	13	49.5 ± 9.8	58.8 ± 4.3	87 ± 1 2.76
$F_{HF_{N}}$	6	61 ± 11.4	43.6 ± 7.1	88.3 ± 9.6
$F_{HF_{W}}$	6	59.2 ± 7	36.1 ± 7.8	144.7 ± 13.3

## 3. Methods

### 3.1. Ethics statement

The study was conducted under ethical approval titled Retrospective analysis of cardiac imaging datasets for development of novel biomarkers study (Short title: RAIDER), approved by GSTT, reference number 306914, protocol version 1.0 01/10/2021.

We developed CEMRG Heartbuilder, a pipeline for the streamlined creation of simulation-ready meshes, generated from a CT scan. The pipeline to process the CT scans is based on the work by [13] but streamlined into a single integrated workflow. Processing of the information consisted of three stages: image to mesh analysis, consisting of a multi-stage segmentation (Section 3.2.1), upsampling, and mesh generation (Section 3.2.2); mesh processing and model creation, which includes the assignment of fibre orientations in the ventricles and atria, and the tagging of the mesh with the different labels needed for the simulations (Section 3.3); and simulations. The multi-stage segmentation and mesh extraction substages are open source, whilst some of the mesh post-processing and simulation stages use the cardiac arrhythmia research package (CARP) [28]. Note that the modular structure of the CEMRG Heartbuilder allows for third-party projects to be included to be made completely open source.

### 3.2. Image to mesh stage

#### 3.2.1 Multi-stage segmentation.

**Fig 2:**
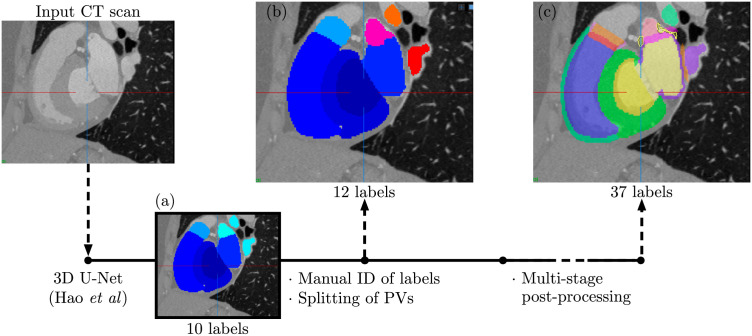
Multi-stage segmentation process. CemrgApp uses the deep learning model developed by [29] to segment the heart. **(a)** The output of the U-Net model, which identifies 10 regions, or “labels”, as described in the text. **(b)** The output of the intermediate stage, where the user manually splits the pulmonary veins into superior and inferior (marked by contrasting colours). **(c)** The final output of the post-processing stage, where the myocardium of the different structures is extracted, increasing the number of labels to 37. The final segmented images are then upsampled to an isotropic resolution of 0.1mm and smoothed.

The image to mesh stage consists of a segmentation of the CT scan via the deep learning model by [29], Fig 2a, which identifies 10 distinct regions: left ventricle myocardium, and blood pools of the left and right ventricles, the left and right atrium, the pulmonary artery, the aorta, the left and right atrial pulmonary veins, and left atrial appendage. Regions are marked in the segmentation with different integer values, we refer to these as “labels” throughout the manuscript. The user then identifies the different labels in the U-Net output, which splits the pulmonary veins into the superior and inferior veins. The user selects two sets of 3 points to identify the location and orientation of the superior and the inferior venae cavae. A post-processing stage follows, increasing the number of labels to 37 (Fig 2b). This stage creates the myocardia, valve planes, and vein rings. The myocardia of the right ventricle (RV), the atria, the aorta and pulmonary artery are extracted through the use of distance maps, with a user-selected thickness of 3.5mm for the RV, and 2mm for the remaining structures and coupling the different regions to ensure no overlap occurs. A table of the labels is provided in S3 Table.

CemrgApp aggregates the different workflows through a combination of code running natively on the app and a docker wrapper, which calls the segmentation and different post-processing stages. The image analysis stage finalises by upsampling the 37-label segmentation to an isotropic resolution of 0.15mm, and a applies a smoothing algorithm [30].

#### 3.2.2. Conversion to mesh.

A volumetric mesh is extracted from the smooth segmentation using a meshing tool developed in-house, which comes packaged with CemrgApp and is based on the Computational Geometry Algorithm Library (CGAL) [31]. The CGAL mesher generates unstructured volumetric meshes composed of linear tetrahedral elements (4 nodes per element). Target element edge length was set to approximately 0.5 mm via the cell_size and facet_size parameters. Mesh density is controlled via CGAL parameters (facet_size, cell_size, facet_distance), which are specified globally for the entire heart geometry. Complete parameter specifications are provided in S2 Table.

**Fig 3:**
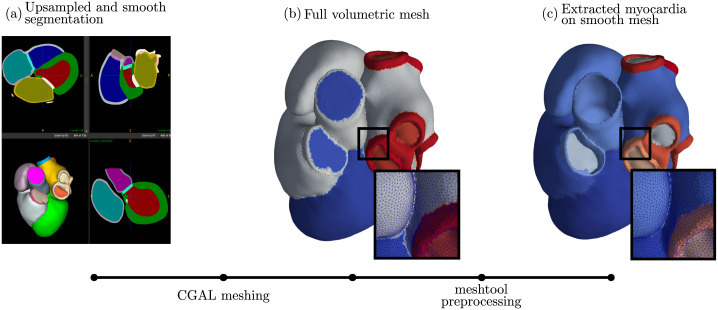
Output of the meshing process from the smooth segmentation to the final working mesh. **(a)** The upsampled and smooth segmentation. **(b)** The mesh extracted from the segmentation using the CGAL meshing tool. It contains all the blood pool and myocardium of the different structures. **(c)** The final mesh after the relabelling, cleaning, smoothing, and extracting only the myocardia and valve planes. Other outputs are created from this final process, such as specific endocardial and epicardial surface meshes for the atria, which will be used later in the process.

The next step is to extract the myocardia, simplify the mesh topology, and relabel the tags using meshtool [32]. The simplification consists in identifying elements insufficiently connected to neighbours of the same class. A complete description of the CGAL parameters, which can be modified by the user, is available in S2 Table. Fig 3 shows the output of the meshing process from the smooth segmentation to the working mesh re-labelled and with the blood pools removed. The final simulation-ready models contained a mean of 2.6 × 10^6^ elements per heart (range 1.9–4.5 × 10^6^).

### 3.3. Mesh processing and model creation

**Fig 4:**
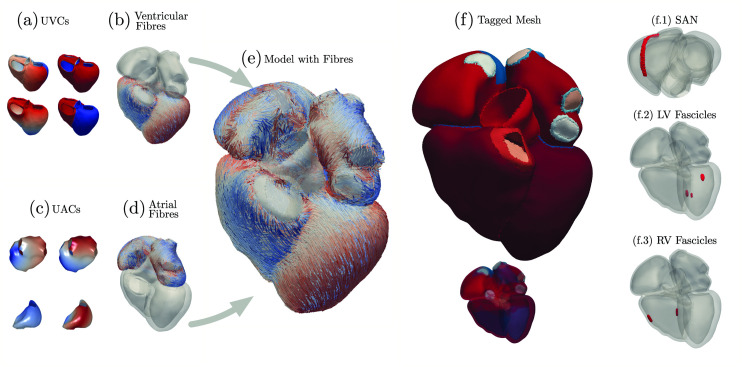
Model creation stage. Universal Ventricular coordinates (a) are calculated from the mesh, then used to assign the fibre direction (b) using a rule-based algorithm [33]. Universal Atrial coordinates (c) are calculated from the mesh, then used to assign the fibre direction (d) using a projection from an atlas [34]. The atrial fibres, which originally are produced on surfaces, are then projected onto the 3D mesh. The fibres from the ventricles and atria are then assigned to the model **(e)**. The final mesh is tagged with the different labels needed for the simulations **(f)**. The fast endocardial conduction layer (FEC) is also created at this stage (f - bottom), which is a thin layer of fast conducting tissue at the endocardium of the ventricles. Finally, UVCs and UACs are used to create the sino atrial node (SAN, f.1), the left ventricle fascicles (f.2) and right ventricle fascicles (f.3).

The last stage of the pipeline included takes the smooth mesh and produces a model with fibre orientations, tagged with the different labels needed for the simulations. This stage is run using CARP and meshtool [28,32] along with the CEMRG Heartbuilder python library. Fig 4 shows a visual overview of the model creation stage, which is described in detail below.

Universal Ventricular Coordinates (UVCs) are a system of coordinates that describe the geometry of the ventricles [35]. Additionally to the ventricles, UVCs are also calculated for the atria, to provide a system of coordinates defined on the volumetric mesh that facilitated the assignment of different tags within the mesh, and of boundary conditions for the pericardium. To achieve this, the left and the right atria were treated as an upside down single ventricle, following the approach described by Strocchi et al. [36], with the apex manually placed between the two right pulmonary veins and behind the supervior vena cava, respectively.

Atrial fibres are then assigned to the mesh by projecting a rule-based fibre field [37] onto the mesh using Universal Atrial Coordinates (UACs) [34]. Ventricular fibres are then assigned to the mesh using a rule-based algorithm, described by [33], which uses a series of Laplace solutions to assign the fibre direction. UACs are a system of coordinates that describe the geometry of the atria [34]. The atrial fibres, which originally are produced on surfaces, are then projected onto the 3D mesh, by assigning endocardial and epicardial fibres in the inner and outer 50% across the thickness of the atria, respectively. From the UAC pipeline, which is run through a docker container, similar to the work by [1], the sino atrial node (SAN) is also mapped from the atlas.

The mesh is tagged with the different labels needed for the simulations. First, a region representing the fast-conducting Bachmann bundle is created, to simulate fast propagation of the electrical signal from the right to the left atrium. This is done using the UVCs defined on the atria. The fast endocardial conduction layer (FEC) is also created at this stage, which is a thin layer of fast conducting tissue at the endocardium of the ventricles representing the Purkinje network. In addition, to prevent unphysiological propagation from the atria to the ventricles and to control the atrioventricular delay, the atria and the ventricles were electrically isolated from each other by defining a layer on non-conducting tissue between the atrial and ventriclar myocardium. Finally, the SAN and the left and right ventricular fascicles are also created, Fig 4 (f.1, f.2, f.3). The SAN and fascicles are used to initiate electrical activation in the electrophysiology (EP) simulations. The left and right fascicles represent the first breakthrough from the Purkinje network into the myocardium to mimic the activation reported by the Durrer maps [38]. These locations are defined in UVCs based on [39].

#### 3.3.1. Mesh quality assessment.

We assessed the quality of all 50 volumetric meshes using the volume-based distortion metric (tet_qmetric_volume) implemented in meshtool [32], which quantifies element distortion relative to an ideal tetrahedron. This metric is directly related to the determinant of the Jacobian of the geometric map and is normalised to [0, 1], where 0 corresponds to a perfect tetrahedron and 1 to a fully degenerate element.

Across all 50 meshes (mean 2.6 × 10^6^ elements per mesh, range $1.9-4.5\times 10^{6}$), the mean element quality was 0.153 ± 0.100 (mean ± SD across all elements in the cohort). The minimum quality per mesh averaged 2.1 × 10^−4^, indicating that even the worst elements retained near-ideal geometry. No inverted elements (quality > 0.99) were detected. Near-degenerate elements (quality > 0.90) were found in 28 of 50 meshes, comprising at most 9 elements per mesh (< 0.0003% of total elements). These results confirm high geometric fidelity suitable for finite element analysis.

### 3.4. Simulations

We performed EP and mechanics simulations on all the models. Simulations were run with a fixed set of parameters to assess the impact anatomical differences would have on the outputs. These simulations also served as an additional quality check for the meshes, to ensure the models could be used to run electromechanics simulations.

Each patient-specific mesh was simulated in its native coordinate system without spatial registration to a common anatomical reference frame. Comparisons between models were performed on scalar outputs (chamber volumes, total activation times) that are invariant to rigid transformations. Universal Ventricular Coordinates (UVCs) [35] provide normalized transmural and apicobasal coordinates for regional functional analysis independent of absolute spatial positioning.

The EP simulations were started at the SAN, defined in Fig 4. The ventricular endocardial electrodes (fascicles) were also activated simulating the early activation sites [40]. The reaction-eikonal model [41] was solved to compute the activation times at each node. A conduction velocity of 1 m/s was used at the myocardial tissue, with a 40% anisotropy cross-fibre, and a 6-fold speed in the fast activation regions [42]. As output, the total activation time of each ventricle, of both ventricles and both atria were extracted.

To test how differences in anatomy impacted simulation predictions, we performed inflation simulations to evaluate the mechanical behaviour of the models. Briefly, increasing pressure is applied at the endocardium of each chamber until a maximum established pressure of 7 mmHg for the left chambers (LV and LA) and 3.5 mmHg for the right chambers (RV and RA) is reached. The myocardium was modelled as an transversely isotropic hyperelastic material using the Guccione’s material law [43], while the rest of the cardiac structures were modelled as isotropic hyperelastic materials using a NeoHookean material law [44]. Mechanics simulations were run with the same boundary conditions as the work by [45], which can be consulted in S1 Text.

Simulations were run to assess the impact of anatomical differences on the outputs and to serve as a quality chech for the meshes. All simulations were run with CARP [28]. The EP simulations were run in a 20-core workstation, while the inflation simulations were run in the UK national supercomputer ARCHER2, using 512 cores.

Model outputs obtained from the simulations are listed in Table 2, along with a brief descripton. The 18 measurements are grouped into four categories: activation times (4 outputs), inflated volume (4), volume change (absolute 4, and normalised 4), and volume ratios (2). The absolute volume change (ΔVol) is calculated as the difference between the maximum and minimum volume during the inflation simulation. For simplicity, we refer to inflated volume as volume throughout the manuscript. The normalised volume change (𝒱) is calculated as the percentage of change in volume, and the volume ratio is calculated as the ratio between the left and right volumes, this was calculated for the ventricles and the atria. The Total Activation Time (TAT) is the time it takes for the electrical signal to propagate through the entire chamber.

**Table 2:** Model outputs obtained from the simulations. The subscript *x* refers to the chamber, which can be left ventricle (LV), right ventricle (RV), left atrium (LA), or right atrium (RA).

Measurement	Symbol	Calculation / Definition
Total Activation Time	${TAT}_{x}$	All chambers, ventricles, and atria
(Inflated) Volume	${Vol}_{x}$	All chambers
Volume Change	$\Delta {Vol}_{x}$	Maximum minus minimum volumes
Normalised Volume Change	${𝒱}_{x}$	Percentage of change in volume
Volume Ratio	$\frac{L}{R}$	Ratio of left and right volumes

### 3.4.1. QRS duration

Since the simulations do not include a torso, the comparison cannot be made directly with the QRS duration measured in the ECGs. Instead, we used the total activation time in the ventricles as a surrogate for QRS duration and compared it between the different groups.

#### 3.4.2. Total activation time in the Atria.

This parameter has emerged as a key marker of atrial remodeling and is associated with *HF* progression and complications [46,47]. We assessed the total activation time in the atria, comparing it between the different groups.

### 3.5. Statistical tests and comparison of volume to clinical literature

We performed a multi‐step statistical analysis of our cardiac dataset. First, we computed descriptive statistics (mean ± SD) for all core metrics (volumes and activation times) stratified by sex, condition (*HF* vs. control), and subtype (*HF*_*N*_ vs. *HF*_*W*_). Differences between narrow and wide QRS durations are only explored qualitatively, as the number of cases in each group is low.

#### 3.5.1. Effect sizes and statistical significance.

To assess the differences in the outcomes, we used a two-way ANOVA to test the effects of Sex, Condition, and their interaction. We followed up significant ANOVA results in the interactions with post-hoc pairwise comparisons using Tukey’s method, and calculated Cohen’s *d* effect sizes [48] for each comparison. If the interaction was not significant, we performed marginal comparisons between *HF* and controls, or male and female groups, across all cases. P‐values were corrected for multiple comparisons using the Benjamini–Hochberg false discovery rate [49]. Effect sizes are reported as Cohen’s *d*, using Gignac’s expanded criteria [50], where *d* < 0.5 is small, 0.5 < *d* < 0.8 is moderate, 0.8 < *d* < 1.2 is large, and *d* > 1.2 is very large.

To externally validate the models, we compared different outputs from the pipeline linked them to a clinically relevant metrics.

#### 3.5.2. Left atrial and left ventricular volume.

Left atrial volume is a predictor of outcome in patients with chronic *HF*, independently of their symptoms, age, or LV function [51]. LV volume is a key prognostic factor in *HF*, as it is associated with the severity of the disease and the risk of adverse events [52,53]. We assessed the left atrial volume (${Vol}_{LA}$) and LV volume (${Vol}_{LV}$) in the models, comparing between the different groups. For ${Vol}_{LV}$, we compared measurements from our cohort with reference values from the UK biobank (UKBB) [54], which defines normal ranges for left and right ventricles in males and females. Outside the normal ranges, we considered the volumes to be abnormal, and therefore indicative of *HF* or other cardiac conditions.

## 4. Results

Fifty whole-heart models (S1 Fig) were created, each including EP and mechanics simulations described in Section 3.4. All simulations were performed with the same parameters for each model. Therefore, the differences in simulations between sexes reported below relate to differences in anatomy. We first examined the distributions of all core metrics across sex and condition. A full table of means and standard deviations is provided in S2 Data. We found no significant difference in age between males and females (M: 53.4 ± 15.0 y; F: 54.6 ± 10.7 y; *p* = 0.75).

### 4.1. Geometric characterization and correlation with simulation outputs

Geometric metrics stratified by sex and condition are provided in S1 Table. Heart failure patients exhibited substantially larger chamber volumes compared to controls, with male HF patients showing the most pronounced dilation (LV: 289 ± 88 mL vs control: 180 ± 36 mL, *p* < 0.001). Myocardial wall volume scaled with anatomical size, ranging from 263 ± 37 cm^3^ in female controls to 396 ± 75 cm^3^ in male HF patients.

Geometric variables showed strong correlations with simulation outputs (S3 Fig). Chamber volume was the strongest predictor of volume change during passive inflation (ρ=0.96 for LV, *p* < 0.001), indicating that larger ventricles exhibit greater absolute compliance under uniform material properties. Myocardial wall volume correlated strongly with activation times (ρ=0.87 for TAT_RV_, ρ=0.85 for TAT_V_, both *p* < 0.001), reflecting the expected increase in conduction path length with anatomical size. Notably, cross-chamber geometric correlations were observed, such as RV volume predicting LV activation time (ρ=0.81, *p* < 0.001), consistent with whole-heart anatomical coupling.

Next, we assessed model volumes and the outputs of the EP and mechanics simulations. In each case, we report the results from the two-way ANOVA and the post-hoc tests, showing the effect sizes. Where no interaction was present, we examined independent effects. In both cases, only statistically significant results after FDR correction are reported here. Effect sizes were computed using Cohen’s *d* and are visualised in Fig 6.

### 4.2. LV volume

**Fig 5:**
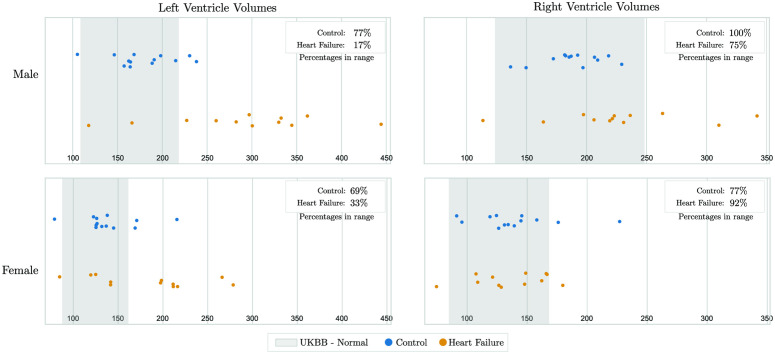
Comparison of measured ventricle volumes from the virtual cohort with clinical literature values and ranges from UKBB. The UKBB normal ranges are defined as measurements within the 95% prediction interval of the study by [54]. Two graphs, corresponding to the LV and RV metrics and reference volumes. Each plot is separated by sex and coloured by condition, with Controls in blue and Heart Failure in orange. Normal ranges are shown as greyed areas, and exceeding the upper limit of the reference range may indicate pathological ventricular dilation. Percentages of each group within the UKBB normal range are displayed in the legend.

**Fig 6:**
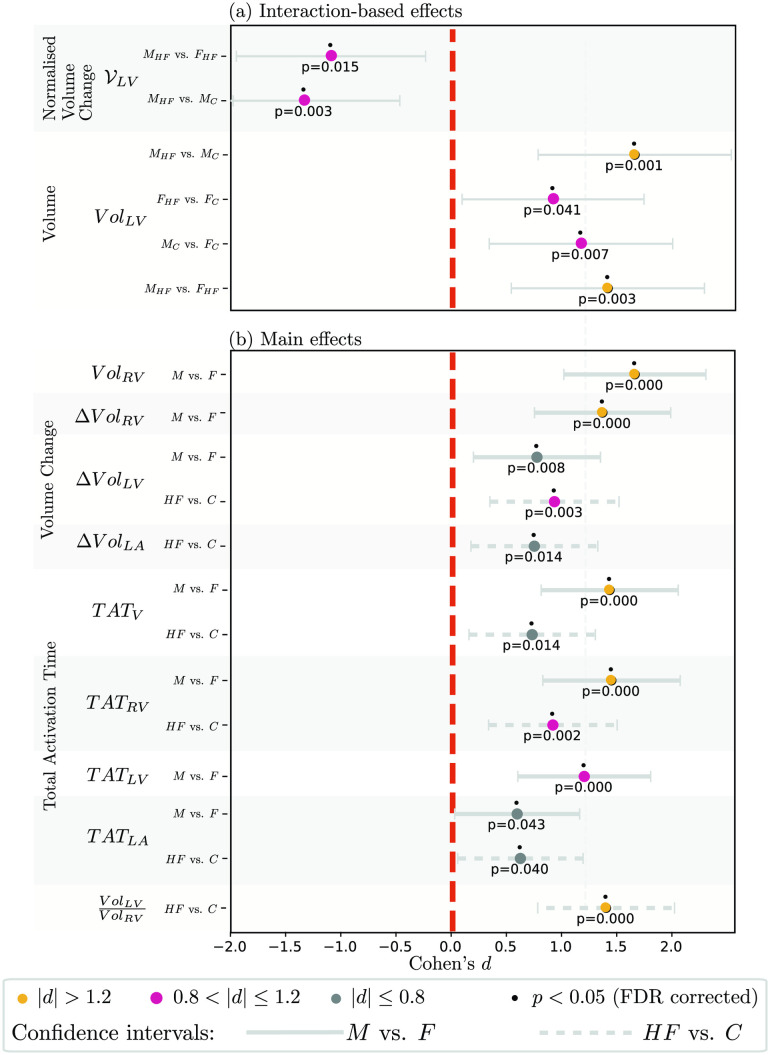
Cohen’s d effect sizes from post-hoc comparisons following two-way ANOVA. Metrics and group comparisons are displayed on the y-axis. This graph only shows effects with a high effect size and a corrected p-value *p* < 0.05. **(a)** Effect sizes for group comparisons performed when the Sex × Condition interaction was statistically significant, justifying 4 simple-effect post-hoc tests. **(b)** Effect sizes for marginal comparisons, when no significant interaction was found. Error bars line styles represent the comparison made: solid for sex, dotted for condition. *Abbreviations*: M = Male, F = Female; C = Control, HF = Heart Failure; TAT = Total Activation time, L/R = Left/Right, V = Ventricle.

${Vol}_{LV}$ was significantly larger in *HF* patients and showed a significant interaction (*p* < 0.05 in ANOVA and all post-hoc comparisons). For all but three male controls, the LV volumes fall within the normal range ([109,218] mL) compared to the UKBB reference values [54]. Similarly, female controls also fall mostly within their corresponding normal range ([88,161] *mL*). We display the results in Fig 5. The effects sizes were also large between *HF* and control groups, but they were much larger in males than in females, for instance, interactions involving females ranged between 0.9 < *d* < 1.17, while interactions involving males were higher than *d* > 1.4 (*p* < 0.05 in all cases, Fig 6(a)).

### 4.3. Volume change in the LV

We used volume change as a surrogate for stiffness. LV volume change was significantly larger in males compared to females and in *HF* patients compared to controls (both main effects with *p* < 0.01), but we did not detect a significant sex × condition interaction (*p* = 0.44), indicating that sex and condition contribute independently to LV volume change. The effect sizes were large, with *d* = 0.94, *p* < 0.01 for *HF* vs controls, and *d* = 0.78, *p* < 0.01 for male vs female comparisons (Fig 6b).

### 4.4. Activation times

For ventricular ${TAT}_{V}$, no significant interaction was found between sex and condition (*p* = 0.76), however, the independent effect sizes were large. First, ${TAT}_{V}$ was significantly longer in males compared to females (*d* = 1.4, *p* < 0.01), and in *HF* patients compared to controls (*d* = 0.72, *p* = 0.014). ${TAT}_{LA}$ showed a similar pattern. First, no significant interaction between sex and condition was found (*p* = 0.24). Second, the independent effects were significant and moderate, showing prolonged activation times in *HF* vs. controls (*d* = 0.62, *p* < 0.05) and prolonged activation in males vs. females (*d* = 0.59, *p* < 0.05).

To assess the validity of using TAT as a surrogate for QRS duration in the absence of torso modeling, we correlated simulated ventricular TAT with recorded ECG QRS duration in cases where ECG data were available (*n* = 33 of 50). In control hearts, TAT showed a positive correlation with QRS duration (Pearson *r* = 0.649, *p* < 0.005, S2 Fig), indicating that simulations using reference conductivity parameters provide an approximate anatomical surrogate for QRS duration, and that anatomical variation captures at least part of the inter-subject variability in activation time. In heart failure patients, no correlation was observed (r=−0.127,p=0.64), consistent with the expected influence of pathological tissue properties (fibrosis, conduction blocks) that are not represented in our standardised parameter set.

### 4.5. Normalised volume changes in LV

${𝒱}_{LV}$ showed a significant interaction in the ANOVA between sex and condition (*p* < 0.05). Post-hoc tests only showed effects sizes were significant when comparing

Male *HF* vs. male controls (d=−1.33),
Male vs. female with *HF* (d=−1.09), both with *p* < 0.05.

## 5. Discussion

We created a sex-balanced virtual cohort of 50 whole-heart models derived from clinical CT scans of both healthy individuals and *HF* patients. We used a semi-automated pipeline to generate these models, which includes segmentation, mesh generation, fibre orientation assignment, and simulation setup. The models enabled the exploration of differences in volume, and activation time across sex and disease groups, using benchmark electrophysiology and mechanics simulations. With this study, we demonstrate that anatomical differences between sexes and across disease states have a significant impact on both electrical and mechanical simulation predictions in the heart. This highlights the critical need to account for such variations when developing and applying computational models in cardiology.

A variety of computational pipelines have been proposed to construct heart models, ranging from atria-only or ventricles-only representations to anatomically detailed four-chamber reconstructions. The works by [55] and [56] proposed patient-specific atrial models, including anatomical regions and fibre orientations from clinical data. In contrast, other pipelines focus only on ventricular chambers [57,58]. A notable whole-heart model was developed by [59], which builds detailed four-chamber meshes of the entire heart from patient imaging and has been enhanced since to improve simulation fidelity, multi-scale integration, and clinical translation. The resulting models are anatomically detailed, but the method depended on extensive user guidance to create the initial atlas, limiting throughput and scale. Other groups, such as Gillette et al. [60], have also demonstrated personalised whole-heart electrophysiology models, but again with significant manual setup and typically small cohorts.

In summary, existing pipelines either produce atria-only or ventricle-only models, or else deliver high-detail four-chamber meshes at the cost of manual effort. Our framework seeks to overcome these limitations by producing anatomically complete four-chamber (atria + ventricles) models with minimal manual input, combining scalability with high anatomical fidelity.

### 5.1. Building patient-specific whole-heart models: Workflow insights

The models were created using a standardised pipeline, CEMRG heartbuilder, which allows for the generation of patient-specific models from CT scans. The segmentation framework has enabled a substantial reduction in overall model generation time relative to earlier methods. The pipeline requires approximately 5 hours per heart on average. It is also significantly more detailed than most publicly available pipelines. For instance, methods evaluated in the MICCAI 2024 Whole Heart Segmentation Challenge [61] typically produce 7–10 output labels, insufficient for physiologically realistic biophysical simulations. In contrast, our approach employs a 37-label standard [13,36], encompassing detailed delineation of blood pools, myocardia, valve planes, and major vessels across all four chambers.

The current implementation applies uniform meshing parameters globally across the whole heart. While regional refinement could theoretically be achieved by meshing structures separately and stitching at interfaces, we prioritized mesh integrity to ensure numerical stability in coupled electromechanical simulations, where interface discontinuities can introduce artifacts.

The strong correlations between geometric metrics and simulation outputs (S1 Table, S3 Fig) demonstrate that anatomical variability is a primary driver of functional differences when using standardized electrophysiological and mechanical parameters. Chamber volume alone explained 92% of variance in volume change during inflation (ρ=0.96), indicating that anatomical size dominates mechanical behavior under uniform constitutive laws. Similarly, myocardial wall volume predicted 76% of variance in right ventricular activation time (ρ=0.87, *p* < 0.001), consistent with total myocardial mass determining conduction path length and duration.

These findings validate our approach of isolating anatomical effects through parameter standardization. However, they also highlight the limitation: real-world patient-specific predictions would require calibration of tissue properties (contractility, stiffness, conduction velocity) to observed clinical metrics.

### 5.2. Impact of anatomical variability on electromechanical predictions

All whole-heart models were simulated using identical parameters for both electrophysiology and mechanics. We did not include active contraction in the benchmark as these simulations often require patient specific parameters to match contraction, pre-load and after load, reducing our ability to compare consistent simulations across anatomies. As such, the results reflect the isolated impact of anatomical structure variation due to sex or disease status on predicted electrical activation and mechanical inflation. The results from the two-way ANOVA showed, in most cases, non significant interactions. This means that we could only look at the independent effects: control vs *HF* and male vs female. The characterisation of effect sizes (small, moderate, and large) by [50], were included as a guideline, based on the distribution of effect sizes obtained.

### 5.3. Comparison with clinical reference ranges

Comparison with population-based MRI reference data [54] demonstrated that control models reproduced expected sex-specific chamber sizes, serving as an anatomical fidelity check for our reconstruction pipeline. Male and female LV and RV volumes were largely consistent with normal ranges, supporting the accuracy of our segmentation and meshing workflow. However, because mechanical simulations used uniform material properties across all cases, the observed volumes represent anatomical structure rather than patient-specific functional state. In heart failure, LV enlargement was most pronounced in males, frequently exceeding reference thresholds, consistent with classical patterns of systolic dilation. Females also showed increased LV volumes, though these remained mostly within upper limits, highlighting sex differences in disease expression. RV enlargement was less prominent in both males and females with HF, reflecting clinical reports that RV size is less sensitive to early disease [62]. These results demonstrate that the virtual cohort captures both healthy anatomy and pathological remodeling patterns at the population level.

### 5.4. Impact of sex and heart failure in *In-Silico* studies

Our simulations consistently separated groups by sex and disease status using uniform electrophysiological and mechanical parameters, demonstrating that anatomical variability alone produces measurable functional differences. When population-level material properties are applied to pathological geometries, the resulting simulations capture group-level trends consistent with clinical observations. Absolute LV volume increases were particularly marked in male HF, reproducing the clinical signature of pathological dilation [52,53]. Larger $\Delta {Vol}_{LV}$ in HF models reflects the mechanical consequence of dilated geometry under uniform compliance parameters [63,64]. Similarly, larger $\Delta {Vol}_{LA}$ patterns align with clinical markers of atrial dysfunction [65,66]. When expressed as normalized change (Fig 6a), however, *HF* females showed the greatest relative differences, driven by smaller baseline chamber sizes. This emphasizes that absolute and relative measures provide complementary insights in sex-stratified analyses [22]. Electrical activation followed similar patterns: ventricular and atrial activation were prolonged in HF and in males, consistent with clinical QRS and conduction observations [46,47]. These convergent trends highlight that population-level simulations with anatomically accurate models can reproduce group-level clinical patterns, while individual patient predictions would require tissue-specific parameter calibration [13].

### 5.5. Establishing model credibility and validation strategies for *In Silico* clinical trials

In silico studies using virtual cohorts of patients, referred to as in silico clinical trials (ISCTs), require careful demonstration of model credibility for their conclusions to be considered reliable. Credibility is primarily established by performing validation, that is, comparison of model predictions with real-world data. However, unlike relatively simple models of standalone medical devices, or drug safety/efficacy studies using a generic action potential model, ISCTs present a much wider range of possible validation strategies. As discussed in detail in [67], possible evaluation activities include: validation of a device/drug model in isolation (e.g., bench test validation of a pacemaker lead mechanics model); individual-level validation of patient models in the cohort (i.e., comparing predictions from a specific patient model with clinical data from the specific patient the model represents), population-level validation of the cohort or sub-cohorts (e.g., comparing the distribution of model outputs across the cohort with clinical data from the corresponding real-world population), validation of coupled device-patient or drug-patient models (e.g., PKPD validation studies), and validation of any statistical model used to convert patient model outputs into clinical endpoints. Also, there are important additional activities that should be performed, such as assessing the virtual cohort representativeness; these are not strictly model validation activities and more akin to model input validation for simple models.

Overall, an ISCT is expected to be supported by a body of evidence on model credibility. For a virtual cohort such as the sex-balanced 50 patient cohort provided here, intended for re-use in variety of future device or drug ISCTs, a hierarchical evaluation approach can be used to generate an initial body of credibility evidence to support a future ISCT.

In this work, we have conducted a preliminary assessment of anatomical representativeness by comparing ventricular and atrial volumes in the male and female cohort subsets with corresponding UK BioBank data. This comparison demonstrates that our reconstruction pipeline accurately captures population-level anatomical distributions, establishing a foundation for future credibility assessments. However, full validation for in silico clinical trials would require additional hierarchical evaluation steps, including patient-specific functional validation, uncertainty quantification, and context-specific device or drug interaction studies [67].

A natural next step would be to perform sex-specific population-level validation: demonstrating that the distribution of model outputs, for each of the male and female sub-cohorts, match the distribution observed in the male and female populations, particularly for outputs for which a statistically significant sex difference was observed. For instance, predicted chamber TAT distributions could be compared with sex-specific population QRS duration data, after an appropriate transformation to account for the fact that TAT is only a surrogate for QRS. Individualized validation, while more challenging, could be performed for newly generated patient models. Ultimately, these results would be supplemented by context-specific validation when the cohort is used in a device/drug ISCT, together with other credibility assessment activities such as verification and uncertainty quantification [68].

### 5.6. Limitations

This work has four main limitations that should be considered when interpreting the results. First, the small sample size within each category of *HF*, narrow and wide QRS duration, limits the statistical power for detecting subtle effects or interactions. Nonetheless, by balancing sex and *HF* subtype, our dataset offers a structured starting point for exploring sex-disease interactions in-silico. Furthermore, it can serve as a foundation for synthetic cohort generation [17,18] with a more diverse range of anatomical and clinical characteristics. Secondly, the simulations performed in this work were benchmark simulations and the parameters were not calibrated to the individual anatomies. However, the use of a common set of parameters across all cases ensured that the differences observed in the results were purely anatomical. Third, the pipeline requires 5 hours per heart on average, with 2 hours spent performing manual corrections required at the segmentation stage. Even so, this is a significant reduction compared to previous methods. Finally, limitations remain at the segmentation stage. Because this step underpins all subsequent model construction, we discuss its implications in more detail in the following section.

Our simulations employed uniform electrophysiological and mechanical parameters across all models to isolate the effects of anatomical variability. The positive correlation between simulated activation times and QRS duration in control hearts (*r* = 0.649, *p* < 0.005) indicates that reference conductivity parameters provide an approximate anatomical surrogate for QRS, and that anatomical variation captures at least part of the inter-subject variability in conduction time. It does not, however, constitute a precise prediction of QRS, nor does it account for patient-specific tissue properties such as fibrosis or conduction system disease. The absence of correlation in heart failure patients (r=−0.127,p=0.64) reflects this limitation and highlights the need for patient-specific parameter calibration.

Additionally, the UKBB reference ranges used for anatomical comparison [54] were derived from a predominantly Caucasian population. Ethnicity data were not systematically collected for the present cohort, which is consistent with common clinical practice in Europe [69], but may limit the generalisability of comparisons with these reference ranges for non-Caucasian patients. Racial differences in cardiac structure and function have been reported [70], underscoring the importance of ethnicity-matched reference data when assessing anatomical fidelity in diverse cohorts.

#### 5.6.1. Critical role of segmentation in model accuracy.

The segmentation stage is a critical bottleneck in the pipeline, requiring notable manual input to ensure anatomical fidelity. The most frequent issues involve small gaps or discontinuities in the myocardium, where the wall becomes excessively thin. The segmentation method proposed by [29] is not robust against some artefacts introduced by implanted devices, such as the bright streaks caused by leads or stents in *HF* patients. Manual steps and corrections in the pipeline could potentially be replaced with more robust algorithms. For example, recent work by [71] demonstrated a foundation model capable of deriving full myocardial masks without user input. Finally, given the limited soft-tissue contrast and resolution of standard CT scans, the myocardial thickness in chambers outside of the LV is set to a user-specified constant. This approximation may not reflect the true myocardial thickness and may underestimate simulation variability.

Full voxel-level correspondence assessment of the 37-label post-processed geometry against clinical images is not feasible for this dataset: CT soft-tissue contrast does not resolve myocardial boundaries outside the LV, and structures including the RV wall, atria, and great vessel walls are assigned thickness via distance maps at a user-specified constant. For the 10-label segmentation stage, geometric accuracy is supported by the published validation of the deep learning model [29]. Population-level consistency of chamber volumes with UKBB reference ranges (Section 3.3) provides indirect geometric plausibility evidence at the cohort level.

#### 5.6.2. Uncertainty quantification.

This study addresses variability across the population — how anatomical differences between patients influence simulation outcomes under fixed parameters. A distinct and equally important source of variability arises during model construction, introduced through segmentation choices and boundary placement. Previous work has begun to quantify this construction-stage uncertainty in the context of left atrial models [1,25], demonstrating that operator variability in segmentation introduces quantifiable differences in chamber geometry and downstream simulation predictions. However, whole-heart models present substantially greater complexity, with four chambers, multiple vessel junctions, and valve planes that may amplify uncertainty propagation through the reconstruction workflow. Explicitly quantifying construction-stage uncertainty in whole-heart models has not been addressed here and represents an important limitation, particularly in the context of regulatory credibility frameworks such as those outlined by the FDA [68].

Despite these limitations, our current approach balances physiological realism with scalability, enabling whole-heart simulations across a sizeable cohort. Incorporating more refined fibre estimation methods remains an important direction for improving predictive accuracy in personalised cardiac models. By incorporating this level of anatomical detail into a reproducible, Python-based workflow, our pipeline bridges the gap between clinical image segmentation and simulation-ready models. It provides a viable route to building whole-heart virtual cohorts for in-silico trials and aligns with regulatory guidelines on model credibility and anatomical completeness [12].

## 6. Conclusion

We presented a cohort of 50 publicly available whole-heart simulations derived from patient-specific CT geometries, comprising healthy individuals and *HF* patients balanced by sex. The dataset offers a representative and well-characterised virtual cohort for in-silico cardiac research. The cohort is suitable for evaluating modeling assumptions, exploring anatomical variability, and help the development of digital-twin–style pipelines contingent on parameter calibration, data assimilation, and validation. Our results show that anatomical variability alone can drive consistent differences between groups, underscoring the need to account for both sex- and disease-specific features when designing and interpreting simulation studies. By combining anatomical fidelity with a reproducible workflow, this work provides a practical foundation for larger-scale investigations and the clinical translation of whole-heart simulations.

## Supporting information

S1 Fig Three-dimensional whole-heart reconstructions from the virtual cohort, grouped and labelled by sex and condition. Each colour corresponds to a different structure in the mesh; each model was derived from a patient’s CT scan and includes fibre orientations, illustrating anatomical variability across sex and heart-failure subtypes. (Originally Fig 5 in main; relocated to supplement per reviewer request.)
(PDF)

S2 FigCorrelation between simulated ventricular total activation time (TAT_*V*_) and recorded ECG QRS duration, stratified by condition.Control hearts (blue, *n* = 17) showed strong positive correlation (Pearson *r* = 0.649, *p* = 0.005), validating TAT_*V*_ as a QRS surrogate when anatomical variability dominates. Heart failure patients (red, *n* = 16) showed no correlation (r=−0.127, *p* = 0.64), reflecting unmeasured pathological tissue properties.(PDF)

S1 Table Geometric characteristics stratified by sex and heart failure status. Mean ± SD reported for age (years), chamber volumes (mL), and total mesh elements (millions).
(XLSX)

S3 FigCorrelation matrix between geometric variables and simulation outputs.Spearman correlation coefficients (ρ) between geometric metrics (chamber volumes, mesh element count, age) and simulation outputs (activation times, volume changes). All shown correlations significant at *p* < 0.05.(PDF)

S2 Table Complete specification of CGAL meshing parameters used for volumetric mesh generation, including facet_size, cell_size, and facet_distance values. (XLSX)

S3 Table List of all 37 anatomical labels assigned during segmentation and mesh generation, including label ID, anatomical structure name, and structure type. (XLSX)

S4 Fig Distribution of mean element quality across the 50-heart cohort. Each point represents one mesh. The tet_qmetric_volume metric ranges from 0 (perfect tetrahedron) to 1 (degenerate); lower values indicate better quality. Cohort-wide mean was 0.153 ± 0.100. No inverted elements (quality > 0.99) were found in any mesh.
(PDF)

S1 Text Detailed specification of pericardial constraints and boundary conditions applied in passive inflation simulations. (TXT)

S1 Data Per-model mesh quality statistics for all 50 hearts. Columns: mesh ID, total element count, min/max/mean/SD of tet_qmetric_volume, number of inverted elements, and percentage of near-degenerate elements.
(CSV)

S2 Data Summary statistics for geometric characteristics and simulation outputs, stratified by sex and heart failure status. (XLSX)

S3 Data Complete ANOVA results for all simulation outputs and geometric metrics. (XLSX)

S4 Data All pairwise post-hoc comparison results (FDR-corrected) for simulation outputs and geometric metrics. Includes Cohen’s *d* effect sizes and 95% confidence intervals.
(XLSX)

S5 Data Descriptive statistics (mean ± SD) for geometric variables stratified by group (Male/Female × Control/HF). (CSV)

S6 Data Spearman correlation coefficients between geometric variables and simulation outputs, with *p*-values and significance levels. (CSV)
