# Supplementary figures and images for: Assessing the importance of sex and disease-specific anatomy in electrophysiology and mechanical simulations with a newly developed public virtual cohort of four-chamber heart models
Source: PLoS Comput Biol. 2026 Jun 2;22(6):e1014325. doi: 10.1371/journal.pcbi.1014325 (PMC13252842; doi:10.1371/journal.pcbi.1014325)

$M_{HFW}$ 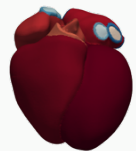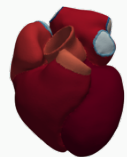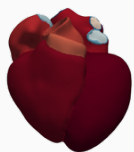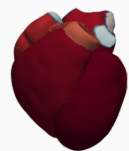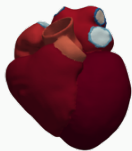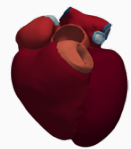 $M_{HFN}$ 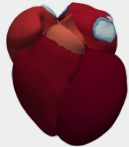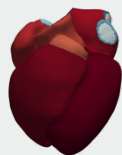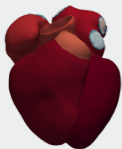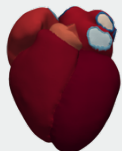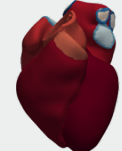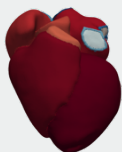 $M_C$ 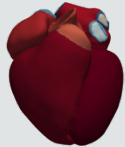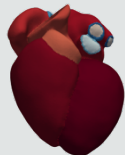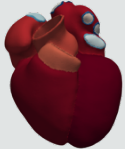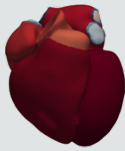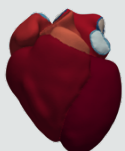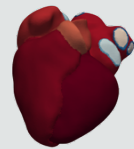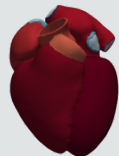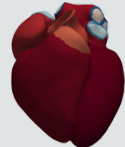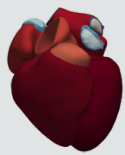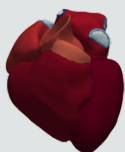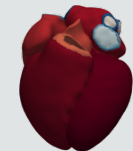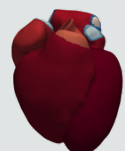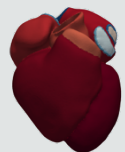 $F_{HFW}$ 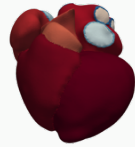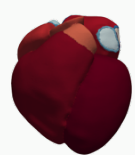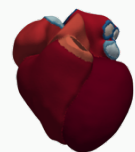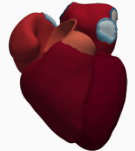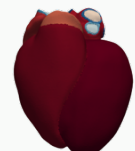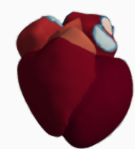 $F_{HFN}$ 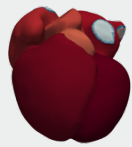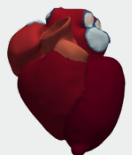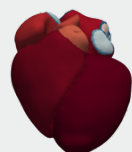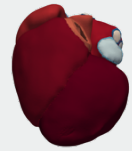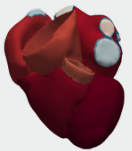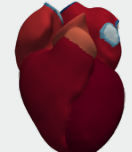 $F_C$ 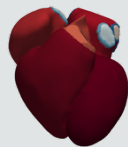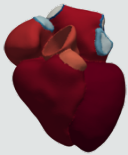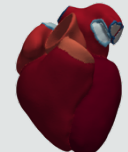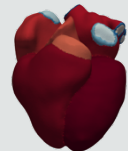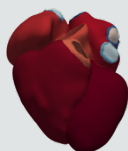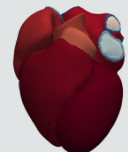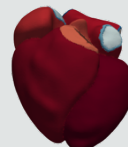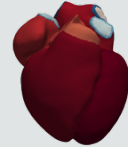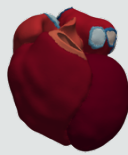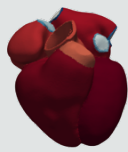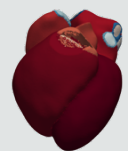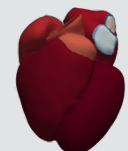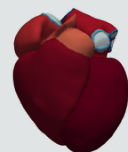

Supplement: S1 Fig — Each colour corresponds to a different structure in the mesh; each model was derived from a patient’s CT scan and includes fibre orientations, illustrating anatomical variability across sex and heart-failure subtypes. (Originally Fig 5 in main; relocated to supplement per reviewer request.) (PDF) [file pcbi.1014325.s001.pdf]

# TAT vs QRS: Stratified by Condition

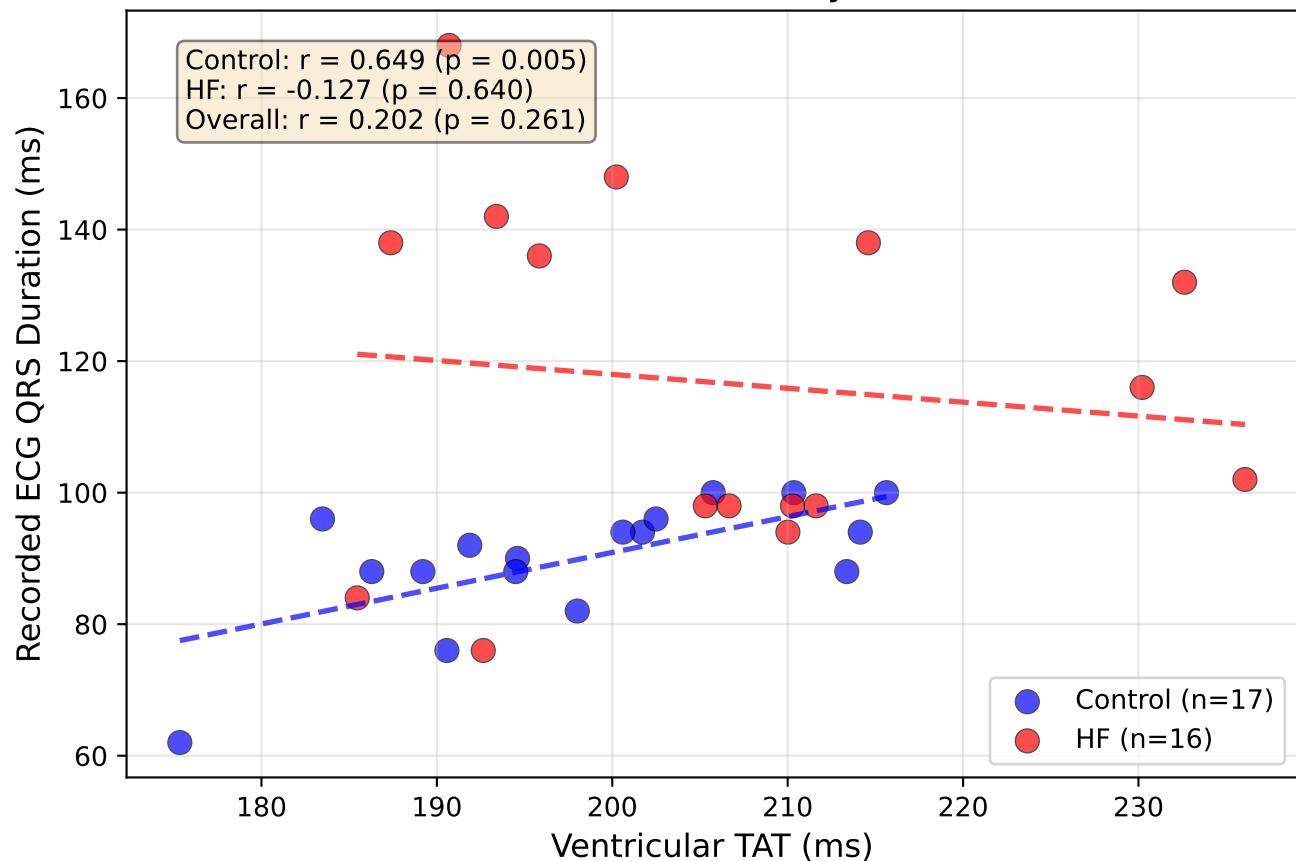

Supplement: S2 Fig — Control hearts (blue, n = 17) showed strong positive correlation (Pearson r = 0.649, p = 0.005), validating TATV as a QRS surrogate when anatomical variability dominates. Heart failure patients (red, n = 16) showed no correlation (r=−0.127, p = 0.64), reflecting unmeasured pathological tissue properties. (PDF) [file pcbi.1014325.s002.pdf]

Correlation: Geometry vs Simulation Outputs

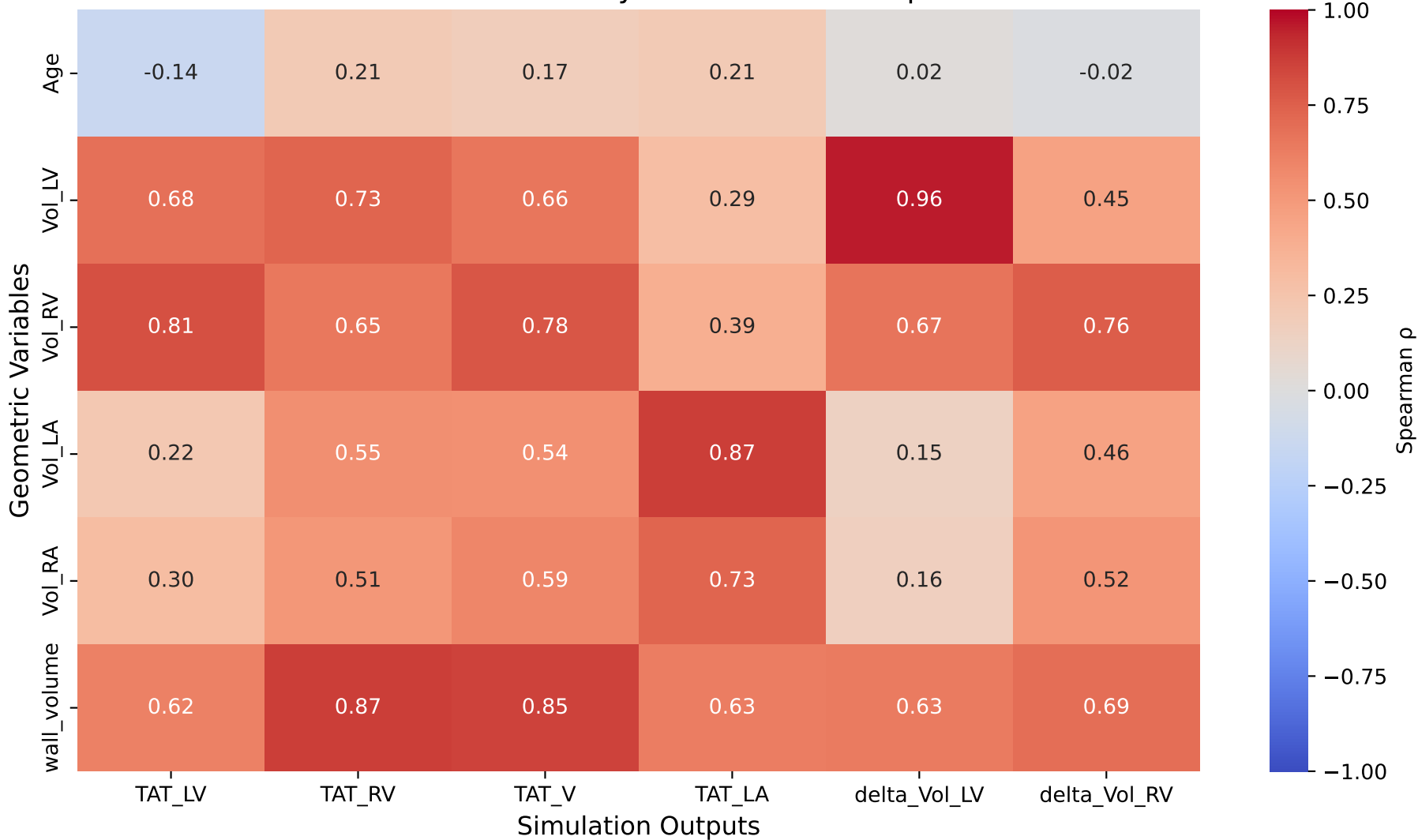

Supplement: S3 Fig — Spearman correlation coefficients (ρ) between geometric metrics (chamber volumes, mesh element count, age) and simulation outputs (activation times, volume changes). All shown correlations significant at p < 0.05. (PDF) [file pcbi.1014325.s004.pdf]

Distribution of Mesh Quality (n=50)

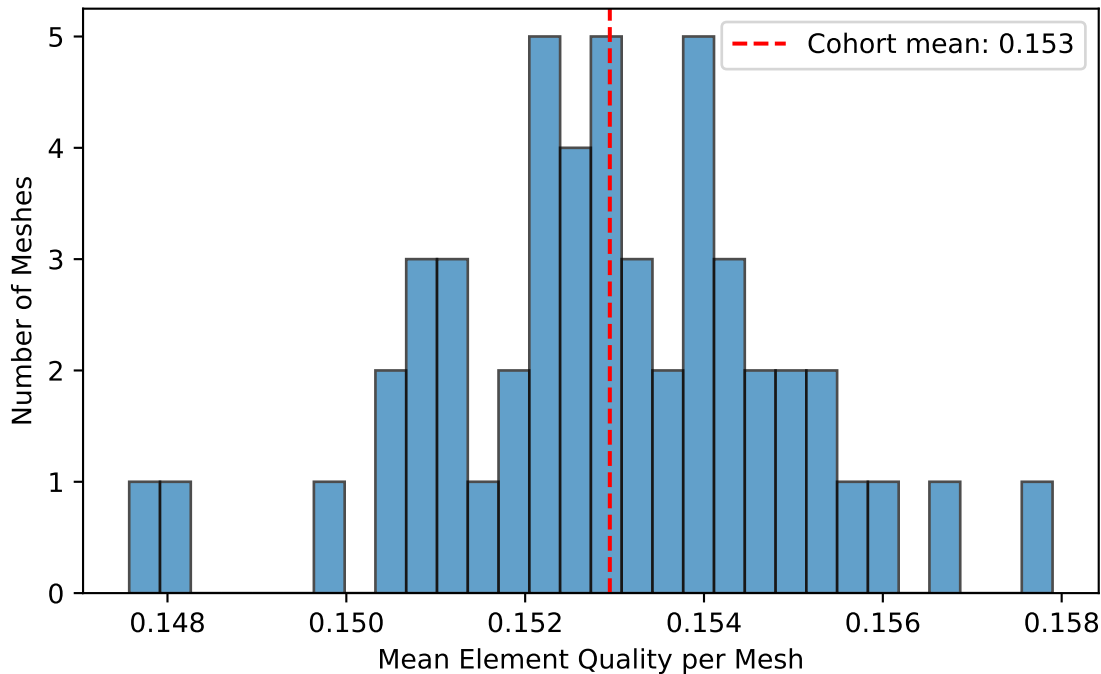

Supplement: S4 Fig — Each point represents one mesh. The tet_qmetric_volume metric ranges from 0 (perfect tetrahedron) to 1 (degenerate); lower values indicate better quality. Cohort-wide mean was 0.153 ± 0.100. No inverted elements (quality > 0.99) were found in any mesh. (PDF) [file pcbi.1014325.s007.pdf]
