# Supplementary material for: Assessing the importance of sex and disease-specific anatomy in electrophysiology and mechanical simulations with a newly developed public virtual cohort of four-chamber heart models
Source: PLoS Comput Biol. 2026 Jun 2;22(6):e1014325. doi: 10.1371/journal.pcbi.1014325 (PMC13252842; doi:10.1371/journal.pcbi.1014325)
Supplement: Attachment — Submitted filename: PCOMPBIOL-D-25-02375_rev.pdf [file pcbi.1014325.s016.pdf]

Manuscript title: Assessing the Importance of Sex and Disease-Specific Anatomy in Electrophysiology and Mechanical Simulations with a Newly Developed Public Virtual Cohort of Four-Chamber Heart Models

Submitted to: PLOS Computational Biology

Reference number: PCOMPBIOL-D-25-02375

The paper presents a cohort of patient-specific virtual models of human hearts suitable for numerical simulations. Starting from medical images, the geometry of the heart is reconstructed via a multi-step process, in which the data are segmented and labeled, and successively, the mesh is created by interfacing with a third-party software (CGAL). The produced dataset is balanced in terms of sex and includes patients with heart disease.

A few clarifications can improve the quality of the manuscript:

1) Are the reconstructed meshes referred to the same timepoint of the cardiac cycle? What is the internal pressure at that instant? Otherwise, how are the models aligned to perform and compare the simulations?
2) Which type of finite elements are supported by the mesh-generator?
3) Have the authors checked the quality of the generated meshes, for instance, by computing the determinant of the Jacobian of the geometrical map? (or the element distortion)
4) Have the authors assessed the quality of the reconstructed geometry compared to the clinical images?
5) Do the users have the possibility of controlling and adapting the mesh by varying the density in specific (user-defined) regions?

Several references are not correctly formatted with respect to the page size; I guess this will be solved during the production stage.
