# Supplementary material for: Assessing the importance of sex and disease-specific anatomy in electrophysiology and mechanical simulations with a newly developed public virtual cohort of four-chamber heart models
Source: PLoS Comput Biol. 2026 Jun 2;22(6):e1014325. doi: 10.1371/journal.pcbi.1014325 (PMC13252842; doi:10.1371/journal.pcbi.1014325)
Supplement: Attachment — Submitted filename: renamed_98ef9.docx [file pcbi.1014325.s015.docx]

Review of “Assessing the Importance of Sex and Disease-Specific Anatomy in Electrophysiology and Mechanical Simulations with a Newly Developed Public Virtual Cohort of Four-Chamber Heart Models” by Solís-Lemus et al.

**Summary:** The authors of the present study present a cutting-edge workflow for the development of image-based cardiac models. The authors construct a sex- and heart-failure-stratified virtual cohorts using data from the UK Biobank, and provide 3D geometries for the four heart chambers, as well as major vessels, using their workflow. The authors then conduct simulations in these geometries under the same model parameterizations. The authors then compare simulation outputs across sex and HF type, and compare their simulation results to the literature.

**Opinion**: This article is a necessary step forward in the development of in-silico clinical trials (ISCTs) and population level models that are necessary for the development of biomedical simulation, digital shadows, and digital twins. The work is novel and impactful, and the authors should be congratulated on this. That being said, there are statements in this manuscript that, I believe, overstep their true impact, as detailed below.

**Major Comments:**

1. Abstract: While the authors later clarify the objectives of the study, the first sentence of the abstract should be more precise since this is the opening information for the article. The study is specifically focused on the effects of anatomical differences on simulation results, and the first sentence should make this clear (e.g., “This work presents a study on how **differences in cardiac anatomy attributed to** sex and disease can influence cardiac electrophysiology and mechanics using a virtual cohort of four-chamber heart models.
2. Section 3.2: “the left and the right atria were treated as an upside down single ventricle” is this based on prior work? If so, please include another citation here to make this clear. If not, how accurate does this methodology work in identifying the atrial geometry?
3. Section 5.3, lines 331-343: While the simulations fall within the MRI reference database, I find it hard to agree that this serves as formal validation. Since the model parameters have been set the same for both sex and heart failure types, the simulated volumes are biased towards these “generalized” parameters. Thus, I think comparisons to clinical references should be done cautiously.
4. Section 5.3, lines 344-364: While I agree that the effect of geometry is definitely influential, as shown here, I don’t believe that this indicates geometry as a definitive factor in altering hemodynamics with HF. We know that afterload, fibrosis, changes in contractility, and other mechanisms develop or adapt simultaneously with HF. So changes in geometry in the absence of different model parameters are likely unphysiological in comparison to subject-specific data, and can’t be extrapolated to a “reason” for differences in hemodynamics alone. I recommend rewriting this paragraph with less emphasis on how geometry leads to differences in HF simulations, and focus more on how “physiological/population level parameters with pathological geometry” generate differences in the model outcomes.
5. Section 5.3 (general): I feel that this article could really provide some foundational insight by focusing more on quantitative differences in geometry with sex and HF type, and then linking this to what is observed in the simulated outputs. For instance, information on how chamber size, artery diameter (since there are 37 regions identified from the segmentation), age, sex, and HF type correlate with “population level” simulations using a common set of parameters might be more useful than directly comparing the model results to sex- and HF-stratified hemodynamic clinical data. The lack of subject-specific (or even group specific) parameterizations makes the current comparisons hard to believe.
6. Section 5.4 (general): I completely agree with the authors that this article is a necessary and refreshing step forward in the generation of ISCT. Again, I think the authors need to be careful in stating that the study is “the first steps of such a hierarchical approach, by assessing cohort representativeness …” If I were to summarize the results, it would suggest that anatomical variability across sex and HF type, without changes in hemodynamic/material parameters, influence electrophysiological and biomechanical simulations. The cohort itself, though, does not represent cardiac function at the level needed for an ISCT, because we know that age, sex, and the presence of HF lead to differences in non-anatomical parameters. This study is important (no doubt about that), but the language used here should be toned down just slightly to make it clear that there is still much work to be done in this space.
7. Section 5.5 and 5.5.1 (general): Since this article shows that anatomical differences with fixed parameters leads to statistically significant differences in simulation results, I believe the authors should discuss the role of uncertainty quantification a bit more here. How would uncertainties in the segmentations and boundaries of the cardiac and vascular geometries contribute to confidence in the simulated outputs? Given this advanced workflow (which we desperately need in our community), it seems like the next necessary step is addressing uncertainty in model geometry, and quantifying this at the forefront.

**Minor Comments:**

1. Figure 2: it is not immediately clear from this figure how subfigure (a) and (b) are different. Consider changing the pulmonary vein colors in subplot (b) to make this more informative.
2. Section 3.2, line 157: “a layer on non-conduting tissue” should be “a layer **of** non-**conducting** tissue”
3. Section 5.2, line 325: please change “structural variation due to sex or disease” to “**anatomical structure** variation due to sex or disease”
