# Supplementary material for: Assessing the importance of sex and disease-specific anatomy in electrophysiology and mechanical simulations with a newly developed public virtual cohort of four-chamber heart models
Source: PLoS Comput Biol. 2026 Jun 2;22(6):e1014325. doi: 10.1371/journal.pcbi.1014325 (PMC13252842; doi:10.1371/journal.pcbi.1014325)
Supplement: Attachment — Submitted filename: response_to_reviewers_update.docx [file pcbi.1014325.s018.docx]

**Response to Reviewers**

*Solis-Lemus et al. | PLOS Computational Biology | PCOMPBIOL-D-25-02375*

We thank the editors and all three reviewers for their careful reading of the manuscript and their constructive comments. We have revised the manuscript extensively in response to the feedback received. Below we address each comment in turn. We submitted a document with tracked changes, where modifications are highlighted in the revised manuscript using the *changes* package, with reviewer-specific colours: Reviewer 1 (blue), Reviewer 2 (orange), Reviewer 3 (teal), and multiple reviewers (magenta).

Line numbers cited below refer to the revised manuscript (without the tracked changes). Where a comment was addressed by changes spanning multiple sections, all relevant locations are noted.

**Note on supplementary material:** New items added during revision are included with this submission: S1 Figure (TAT–QRS correlation), S1 Table (geometric characteristics), S2 Figure (correlation heatmap), S2 Table (CGAL parameters), S3 Figure (mesh quality distribution), and data files S1–S6. Pre-existing items S3 Table (37 labels), S1 Text (boundary conditions), and associated statistical data files are also included. Numbering follows the order listed in the Supporting Information section of the revised manuscript.

**Response to Editor**

**E1: CRediT author contributions**

**Reviewer comment:** *Please ensure that the CRediT author contributions listed for every co-author are completed accurately and in full.*

CRediT contributions have been completed in full in the online submission system for all 18 co-authors listed by the editor. The contribution taxonomy from https://journals.plos.org/ploscompbiol/s/authorship#loc-author-contributions was used.

**Changes made:** Updated in the online submission form; author contribution statements also added to the manuscript.

**E2: Manuscript source file**

**Reviewer comment:** *Please upload your manuscript file as a .doc, .docx, .rtf or .tex.*

A .tex export of the revised manuscript is included with this submission.

**Changes made: A** single .tex file with the entire manuscript has been uploaded to the submission system.

**E3: Supporting Information legends missing**

**Reviewer comment:** *You have not included a list of legends. Please add a full list of legends for your Supporting Information files after the references list.*

A complete Supporting Information section has been added after the References list, containing legends for all supplementary items: Figures S1-S3, Tables S1-S3, S1 Text, Data files S1-S6.

**Changes made:** Added Supporting Information legends section after References in revised manuscript.

**E4: Figure 1 copyright/clip-art**

**Reviewer comment:** *Please confirm whether you drew the images / clip-art within the figure panels by hand.*

The heart icons in Figure 1 were derived from a CC0 image sourced from openclipart.org (<https://openclipart.org/246884>) and modified to fit the figure design.

**Changes made:** Copyright confirmation provided. Figure 1 only changed the heart icons.

**E5: Data availability**

**Reviewer comment:** *All PLOS journals now require all data underlying the findings to be freely available.*

The virtual cohort is publicly available at https://doi.org/10.5281/zenodo.17048090 as stated in the abstract and manuscript. No ethics breach applies; the original CT data are covered by ethical approval (RAIDER, GSTT reference 306914). The cohort models and simulation outputs are the data underlying the findings, and these are freely available at the Zenodo repository.

**Changes made:** Data availability statement confirmed in submission form.

**E6: Financial Disclosure**

**Reviewer comment:** *Please amend your detailed Financial Disclosure statement.*

The Financial Disclosure has been updated to full sentences in the submission form: the funders had no role in study design, data collection and analysis, decision to publish, or preparation of the manuscript. No authors received salary from the funders for this study.

**Changes made:** Financial Disclosure updated in submission form.

**E7: File ordering**

**Reviewer comment:** *Please ensure that the files are uploaded in the online submission form in a correct numerical order.*

All files have been renumbered and re-uploaded in correct numerical order.

**Changes made:** File order corrected in submission system.

**Response to Reviewer 1**

**R1-Maj-1: Abstract Opening Sentence**

**Reviewer comment:** *The study is specifically focused on the effects of anatomical differences on simulation results, and the first sentence should make this clear (e.g., 'This work presents a study on how differences in cardiac anatomy attributed to sex and disease can influence cardiac electrophysiology and mechanics...').*

We thank the reviewer for this suggestion. We have revised the abstract opening sentence to explicitly state that the study focuses on anatomical differences attributed to sex and disease, adopting the reviewer's proposed wording.

**Changes made:** Abstract line 1 revised to: ‘This work presents a study on how differences in cardiac anatomy attributed to sex and disease can influence cardiac electrophysiology and mechanics using a virtual cohort of four-chamber heart models.’

**R1-Maj-2: Upside-Down Ventricle Citation**

**Reviewer comment:** *Section 3.2: 'the left and the right atria were treated as an upside down single ventricle' — is this based on prior work? If so, please include another citation here.*

We thank the reviewer for this suggestion. The method outlined by the reviewer has previously been utilised in our research group. We have now added an explicit citation to Strocchi et al. and clarified that the process is purely geometric, facilitating the assignment of transmural and apicobasal coordinates for the atria.

**Changes made:** Citation added to Methods Section 3.2 [page 6, lines 144-145].

**R1-Maj-3: MRI Comparison Is Not Formal Validation**

**Reviewer comment:** *Section 5.3 lines 331-343: I find it hard to agree that this serves as formal validation. Since the model parameters have been set the same for both sex and heart failure types, the simulated volumes are biased towards these 'generalized' parameters.*

We thank the reviewer for this important clarification. We have reframed the UKBB comparison throughout as an 'anatomical fidelity check' rather than functional validation. We now explicitly state that because mechanical simulations used uniform material properties across all cases, the observed volumes represent anatomical structure rather than patient-specific functional state. This comparison validates our reconstruction pipeline's ability to capture population-level anatomical distributions, not patient-specific hemodynamic function.

In addition, we correlated simulated ventricular total activation time (TAT) with recorded ECG QRS duration (n=33 of 50 cases with available ECG data). In control hearts, TAT showed a positive correlation with QRS duration (Pearson r = 0.649, p = 0.005), suggesting that anatomical variation contributes to the observed association between TAT and QRS duration. We interpret this analysis cautiously: it supports the view that anatomy contributes to inter-subject variation in activation timing, but it does not replace formal validation against matched patient-specific functional measurements. This result is presented in the Results section and Supplementary Figure S1.

**Changes made:** Discussion Section 5.3 rewritten: 'validation' replaced with 'anatomical fidelity check'; explicit statement on uniform material property limitation added [pages 15-16, lines 396-410]. TAT-QRS correlation added to Results [page 13, lines 329-338] and Limitations [page 17, lines 489-496].

**R1-Maj-4: Geometry Causality Overstated**

**Reviewer comment:** *Section 5.3 lines 344-364: I don't believe that this indicates geometry as a definitive factor in altering hemodynamics with HF. Changes in geometry in the absence of different model parameters are likely unphysiological in comparison to subject-specific data.*

We thank the reviewer for this critical feedback. We have substantially revised this paragraph to eliminate causality claims. The revised text explicitly frames our findings as demonstrating what happens 'when population-level material properties are applied to pathological geometries', rather than claiming geometry alone drives hemodynamic changes. We state clearly that individual patient predictions would require tissue-specific parameter calibration, while our results show that population-level simulations with anatomically accurate models reproduce group-level clinical patterns.

**Changes made:** Discussion Section 5.3, paragraph 'Impact of Sex and Heart Failure in In-Silico Studies': complete rewrite removing geometry-to-hemodynamics causality language [page 16, lines 411-429].

**R1-Maj-5: Quantitative Geometric Characterisation**

**Reviewer comment:** *I feel that this article could really provide some foundational insight by focusing more on quantitative differences in geometry with sex and HF type and then linking this to what is observed in the simulated outputs.*

We thank the reviewer for this suggestion. Following reviewer feedback, we have replaced mesh element count with myocardial wall volume (cm³) as a geometric predictor, as element count is mesh-resolution-dependent rather than a biological quantity. Myocardial wall volume showed strong correlations with activation times (ρ=0.87 for TAT_RV, ρ=0.85 for TAT_V, both p<0.001) and moderate correlations with volume change (ρ=0.69 for ΔVolRV, ρ=0.63 for ΔVolLV, both p<0.001), indicating that total myocardial mass is a primary anatomical driver of both electrical and mechanical simulation outputs.

**Changes made:** Added: Supplementary Table S1 (geometric characteristics by sex/condition); Supplementary Figure S2 (correlation heatmap). Added Results paragraph on geometric-functional correlations [page 11, lines 269-283]. Added Discussion paragraph interpreting these findings [page 15, lines 370-382].

**R1-Maj-6: ISCT Language De-escalation**

**Reviewer comment:** *Section 5.4: the language used here should be toned down just slightly to make it clear that there is still much work to be done in this space.*

We thank the reviewer for this perspective. We have revised Section 5.4 to replace *'first steps of such a hierarchical approach'* with *'preliminary assessment of anatomical representativeness'*. We now explicitly acknowledge that full validation for in silico clinical trials would require additional hierarchical evaluation steps, including patient-specific functional validation, uncertainty quantification, and context-specific device or drug interaction studies. We have cited Pathmanathan et al. (2024) to provide the appropriate ISCT credibility framework.

**Changes made:** Discussion Section 5.4: de-escalated ISCT language, added explicit acknowledgment of required future validation steps, cited Pathmanathan 2024 [page 17, lines 454-461].

**R1-Maj-7: Uncertainty Quantification**

**Reviewer comment:** *Section 5.5 and 5.5.1: I believe the authors should discuss the role of uncertainty quantification a bit more here. How would uncertainties in the segmentations and boundaries contribute to confidence in the simulated outputs?*

We thank the reviewer for this suggestion. We have added a new Section 5.5.2 on Uncertainty Quantification, aiming to more clearly distinguish between two sources of variability. This study addresses variability across the population, that is, how anatomical differences between patients influence simulation outcomes. A separate and important source of variability arises during model construction itself, introduced through segmentation choices and boundary placement. This construction-stage uncertainty has been addressed in previous work through approaches such as [Solís-Lemus et al., 2023; Sim et al., 2019], but has not been explicitly quantified here. We acknowledge this as a limitation, particularly in the context of regulatory credibility frameworks.

**Changes made:** New Discussion Section 5.5.2 'Uncertainty Quantification' added [page 19, lines 577-600].

**R1-Min-1: Figure 2 Visual Distinction**

**Reviewer comment:** *Figure 2: it is not immediately clear from this figure how subfigure (a) and (b) are different. Consider changing the pulmonary vein colors in subplot (b).*

We thank the reviewer for this suggestion. We have revised Figure 2 to use contrasting colors (magenta, orange, red) for the pulmonary vein regions in subplot (b), clearly differentiating the manually split superior and inferior components from the original segmentation shown in panel (a). A note has been added to the figure caption.

**Changes made:** Figure 2 panel (b) recolored; caption updated.

**R1-Min-2: Typographical Error: 'non-conduting'**

**Reviewer comment:** *Section 3.2, line 157: 'a layer on non-conduting tissue' should be 'a layer of non-conducting tissue'.*

We thank the reviewer. This has been corrected.

**Changes made:** Methods Section 3.2: corrected to 'a layer of non-conducting tissue' [page 7, line 164].

**R1-Min-3: Wording: 'structural variation'**

**Reviewer comment:** *Section 5.2, line 325: please change 'structural variation due to sex or disease' to 'anatomical structure variation due to sex or disease'.*

We thank the reviewer. This has been corrected.

**Changes made:** Discussion Section 5.2: corrected to 'anatomical structure variation' [page 15, line 390].

**Response to Reviewer 2**

*Reviewer 2 raised five technical questions, all of which require specific numerical or methodological answers. Each is addressed below with the exact data from our pipeline. These were the highest-priority items in the revision.*

**R2-1: CT Acquisition Phase and Internal Pressure**

**Reviewer comment:** *Are the reconstructed meshes referred to the same timepoint of the cardiac cycle? What is the internal pressure at that instant?*

We thank the reviewer for this question. All CT scans were acquired using a consistent imaging protocol across the cohort. The reconstructed meshes represent the cardiac anatomy in a static reference configuration. While the scans are not ECG-gated to a specific cardiac phase, the use of a uniform protocol supports comparative analysis at the population level, although it does not provide phase-matched subject-specific anatomy.

Our simulation framework applies prescribed endocardial pressures (7 mmHg for left chambers, 3.5 mmHg for right chambers) from this baseline geometric state, enabling direct comparison of how anatomical differences attributed to sex and disease influence mechanical response under identical loading conditions.

**Changes made:** Added clarification to Materials Section 2 regarding CT acquisition protocol and reference configuration [3, lines 69-73].

**R2-2: Finite Element Type**

**Reviewer comment:** *Which type of finite elements are supported by the mesh-generator?*

We thank the reviewer for this question. The CGAL mesh generator produces unstructured volumetric meshes composed of linear tetrahedral elements (4 nodes per element), with target element edge length set to approximately 0.5 mm. After all model processing steps (fibre assignment, coordinate mapping, and region tagging), the final simulation-ready models contained a mean of 2.6×10⁶ elements per heart (range 1.9–4.5×10⁶). Complete CGAL parameter specifications are provided in Supplementary Table S2.

**Changes made:** Added element type specification and final mesh statistics to Methods Section 3.1.2 [pages 5-6, lines 117-132].

**R2-3: Mesh Quality Assessment**

**Reviewer comment:** *Have the authors checked the quality of the generated meshes, for instance, by computing the determinant of the Jacobian of the geometrical map?*

We thank the reviewer for this important question. We have now assessed mesh quality using the volume-based tetrahedral distortion metric (tet_qmetric_volume) implemented in **meshtool**, which is directly related to the Jacobian determinant of the geometric map. We included Figure S3 in the supplementary material, showing the distribution of the distortion metric across all meshes in the cohort and Supplementary Data S8, a CSV file with all the outputs from this analysis.

Across all 50 meshes (mean 2.6×10⁶ elements per mesh), the mean element quality was 0.153 ± 0.100 on a normalised scale where 0 represents a perfect tetrahedron and 1 represents full degeneracy. The minimum quality per mesh averaged 2.1×10⁻⁴, indicating that severely distorted elements were rare. Critically, no inverted elements (quality > 0.99) were detected in any mesh, and near-degenerate elements (quality > 0.90) were found in only 28 of 50 meshes, comprising at most 9 elements per mesh (< 0.0003% of total elements).

All simulations converged without numerical instability, providing additional functional confirmation of mesh suitability.

**Changes made:** Mesh quality assessment added to Methods Section 3.1.2 [page 7, lines 171-182].

**R2-4: Geometry-Image Correspondence and Model Alignment**

**Reviewer comment:** *Have the authors assessed the quality of the reconstructed geometry compared to the clinical images? How are the models aligned to perform and compare the simulations?*

We thank the reviewer for this two-part question.

Regarding model alignment: each patient-specific mesh was simulated in its native coordinate system without spatial registration to a common anatomical reference frame. This approach is appropriate because all comparisons are performed on scalar outputs (chamber volumes, total activation times, volume changes) that are invariant to rigid spatial transformations. Universal Ventricular Coordinates (UVCs) provide normalised transmural and apicobasal coordinates for any regional functional analysis, enabling comparison independent of absolute spatial positioning. This has been clarified in Methods Section 3.3.

Regarding geometry-image correspondence: full voxel-level correspondence assessment of the 37-label post-processed geometry against clinical images is not feasible for this dataset for two reasons. First, CT soft-tissue contrast does not resolve myocardial boundaries outside the LV; structures including the RV wall, atria, and great vessel walls are assigned thickness via distance maps at a user-specified constant, and the SVC and IVC are not visible in standard clinical CT scans. Second, there is no independent ground-truth segmentation available. For the 10-label segmentation stage, geometric accuracy is supported by the published validation of the deep learning model (Xu et al.). Population-level consistency of chamber volumes with UKBB reference ranges (Section 5.3) provides indirect geometric plausibility evidence at the cohort level. These constraints are acknowledged as a limitation in Discussion Section 5.5.1.

**Changes made:** Spatial alignment clarification added to Methods Section 3.3 [page 9, lines 188-193]. Geometry-image correspondence limitation added to Discussion Section 5.5.1 [page 18, lines 516-523].

**R2-5: User Control of Mesh Density**

**Reviewer comment:** *Do the users have the possibility of controlling and adapting the mesh by varying the density in specific (user-defined) regions?*

We thank the reviewer for this question. Mesh density is controlled via CGAL parameters (facet_size, cell_size, facet_distance), which are specified globally for the entire heart geometry in the current implementation. While regional refinement is theoretically possible by meshing structures separately and stitching at interfaces, we prioritised maintaining mesh integrity and element quality consistency across the whole heart to ensure numerical stability in coupled electromechanical simulations, where interface discontinuities can introduce artefacts. Complete parameter specifications are provided in Supplementary Table S2. The trade-offs of regional refinement are discussed in the Workflow Insights subsection.

**Changes made:** Mesh control documentation added to Methods Section 3.1.2 [page 5, lines 119-125]. Discussion of regional refinement trade-offs added to Discussion Section 5.1 [page 15, lines 366-370].

**Response to Reviewer 3**

**R3-Maj-1: Introduction Structure and Tense**

**Reviewer comment:** *The last two paragraphs of the introduction section are too long. I would say the very last paragraph is not needed. The second to last paragraph has too much methodology for an intro. Also, these two paragraphs jump tenses too much.*

We thank the reviewer for this feedback. We have streamlined the introduction as follows:

1. Last paragraph: we removed the redundant aims statement ('The aim of this study is to develop a workflow...') as these objectives are already clear from the preceding paragraph. Only the reference to Figure 1 is retained.
2. Second-to-last paragraph: we removed methodological details, eliminated sample size numbers (n=26, n=12, n=12), and reduced length by approximately 50%. Technical details have been moved to the Methods section.
3. Tense consistency: both paragraphs have been revised to use consistent past tense throughout.

**Changes made:** Introduction final paragraph: aims statement deleted [see changes document]. Second-to-last paragraph: streamlined, sample sizes removed, tense corrected [page 3, lines 53-63].

**R3-Maj-2 / R3-Maj-6: Lack of Proper Validation**

**Reviewer comment:** *The main issue with the paper is lack of proper validation. There is comparison of lung volumes and ephys between groups, but there is no validation back to patient data. Section 5.3 — given the point above, this was not a convincing argument.*

We thank the reviewer for this comment. We agree that the original text overstated the extent to which the present analyses could be interpreted as validation against patient data, and we have revised the manuscript to correct this. In the revised version, we make a clearer distinction between two separate questions: whether the virtual cohort is anatomically plausible at the population level, and whether the simulations achieve patient-specific predictive accuracy. The present study addresses the former, but not the latter. Our objective here is to examine how differences in anatomical geometry across sex and heart failure groups affect simulated outputs under a common set of model assumptions, rather than to reproduce each patient’s physiology through case-specific calibration. Accordingly, the comparison to UK Biobank reference ranges (Section 5.3) is now described as an anatomical fidelity check rather than formal validation. We now show in Fig. 6 the proportions of cases within UKBB reference ranges per group, providing quantitative context for the anatomical comparison.

We have also added a limited physiological consistency analysis comparing simulated ventricular total activation time with recorded QRS duration in the subset of cases with ECG data. In control hearts, these measures were positively correlated (Pearson r = 0.649, p = 0.005), suggesting that anatomical variation contributes to inter-subject differences in activation timing under common conductivity assumptions. In HF patients, no correlation was found (r = -0.127, p = 0.64), consistent with unmeasured pathological tissue properties, which is acknowledged as a limitation. We interpret that result conservatively: it indicates that anatomy contributes to observed inter-subject variation in activation timing, but it does not establish patient-specific validation. We now state explicitly that rigorous predictive validation would require matched functional measurements, gated imaging, and patient-specific parameter estimation, which were not available in this retrospective dataset.

**Changes made:** Discussion Section 5.3 reframed [pages 15-16, lines 396-410]. TAT-QRS analysis added to Results [page 13, lines 329-338] and Limitations [page 17, lines 489-496]. Figure 6 revised with percentage annotations.

**R3-Maj-3: QRS Duration Without Full Torso**

**Reviewer comment:** *There should be a way to get at an estimate for the QRS duration without having to model the full torso. This is really needed to allow the work to be validated against physiological data.*

We thank the reviewer for this constructive suggestion. We have now performed a correlation analysis between simulated ventricular total activation time (TAT) and recorded ECG QRS duration in cases with available ECG data (n=33 of 50). In control hearts, TAT showed a strong positive correlation (r = 0.649, p = 0.005), suggesting that anatomical variation contributes to observed variation in depolarisation timing under common conductivity assumptions. In heart failure patients, no correlation was observed (r = -0.127, p = 0.64), consistent with the known influence of pathological tissue properties not represented in our standardised parameter set. We interpret this analysis as a limited physiological consistency check rather than formal validation. The result is presented in the Results section and illustrated in Supplementary Figure S1.

**Changes made:** TAT-QRS correlation analysis added to Results [page 13, lines 329-338]. Limitation paragraph added to Discussion Section 5.5 [page 17, lines 489-496]. Supplementary Figure S1 added.

**R3-Maj-4: Figure 5 Clarity**

**Reviewer comment:** *Fig 5 is not informative. All I can see are columns of images that to my eye look completely identical. If the reader is supposed to see differences they need to be properly blown up and pointed out. Alternatively, move this to the supplemental if it is not essential.*

We thank the reviewer for this feedback. We agree that Figure 5 is not intended to support quantitative anatomical comparison between individual cases. Its purpose is to provide a cohort-level overview of the full set of simulation-ready whole-heart models generated by the workflow. To make this clearer, we have revised the caption accordingly and ensured that the quantitative comparisons are presented in the main text and supplementary analyses.

**Changes made:** Figure 5 revised: We have added a sentence to the Figure 5 caption clarifying its purpose as a cohort overview.

**R3-Maj-5: Figure 6 Quantitative Validation**

**Reviewer comment:** *Looking at the data in Fig 6, this does not look like a proper validation. There are plenty of blue (control) dots outside the gray band. And plenty of red dots inside the gray band. If this is to be taken as validation it is very qualitative.*

We thank the reviewer for this important feedback. We have revised Figure 6 to include quantitative annotations showing the percentage of cases falling within the UKBB reference ranges for each panel:

- LV controls: 77% (males) and 69% (females) within normal ranges.
- LV in HF: 17% (males) and 33% (females) within normal ranges, which is consistent with pathological ventricular dilation.
- RV controls: 100% (males) and 77% (females) within range.
- RV in HF: 75–92% within range, reflecting that RV enlargement is less prominent than LV dilation.

We have also added these percentages as text in the Results section and clarified in Section 5.3 that this comparison serves as an anatomical fidelity check, not functional validation.

**Changes made:** Figure 6 revised with percentage annotations. Discussion Section 5.3 updated.

**R3-Maj-7: UKBB Caucasian Reference Ranges**

**Reviewer comment:** *Line 233 states that the UK Biobank defines the reference ventricle volumes. It is unclear whether this definition that was based on only Caucasians is an appropriate standard for the patient demographics in this study.*

We thank the reviewer for raising this important limitation. We have added a paragraph to the Limitations section (5.5) acknowledging that the UKBB reference ranges were derived from a predominantly Caucasian population (Petersen et al., 2017). Ethnicity data were not systematically collected for this cohort, consistent with common clinical practice in Europe (Sheikh et al., 2004), but this may limit generalisability for non-Caucasian patients. Racial differences in cardiac structure have been reported (LaBounty et al., 2019), underscoring this limitation.

**Changes made:** Limitations Section 5.5: new paragraph on UKBB Caucasian-centric reference ranges added [page 17, lines 497-503].

**R3-Min-1: Figure 7b Line Distinction**

**Reviewer comment:** *In figure 7b, it is quite difficult to distinguish which line of comparison corresponds to which parameter.*

We thank the reviewer. Figure 7b has been revised to use distinct line styles: solid lines for sex comparisons (M vs F) and dotted lines for condition comparisons (HF vs C). Line weight has been increased and a legend indicating the line style convention has been added to the figure. A note on this convention has been added to the figure caption.

**Changes made:** Figure 7b revised: distinct line styles, increased line weight, line style legend added. Caption updated.

**R3-Min-2: Figure 7a Reference Ambiguity**

**Reviewer comment:** *Lines 350-352, is this referencing figure 7a? It is unclear whether 'HF females showed the greatest relative differences' is in comparison to HF males or to control females.*

We thank the reviewer for noting this ambiguity. The sentence has been revised to read: 'When expressed as a normalised change (Figure 7a), HF females showed the greatest relative differences compared to HF males, a finding likely driven by smaller baseline chamber sizes.'

**Changes made:** Discussion Section 5.3: sentence clarified to specify comparison group and Figure 7a reference [page 16, line 421].

**R3-Min-3: CM&S Acronym**

**Reviewer comment:** *The acronym CM&S is not necessary and makes it harder to read the paper. Just use the words.*

We thank the reviewer. All instances of 'CM&S' have been replaced with 'computational models and simulations' throughout the manuscript.

**Changes made:** All CM&S instances removed across introduction, methods, and discussion.

**R3-Min-4: Citation Numbers Used as Nouns**

**Reviewer comment:** *Citation numbers are used as nouns, which makes reading the paper awkward. Reading '[13] did this' is very strange.*

We thank the reviewer. We have scanned the full manuscript and revised all instances where citation numbers were used as sentence subjects. For example, 'The cohorts of cases presented by [13]...' has been revised to 'The cohort presented by Strocchi et al. [13]...', and similarly throughout the Introduction and Discussion.

**Changes made:** Citation-as-noun instances corrected in Introduction [lines 31, 44] and Discussion.

**R3-Min-5: HF Spacing Error**

**Reviewer comment:** *Line 261 HF has strange spacing.*

We thank the reviewer. The spacing inconsistency in the HF macro at that line has been corrected.

**Changes made:** HF spacing fixed in Results.

**Supplementary Material — Status Summary**

The following supplementary items are included with this submission. Items marked NEW were added in response to reviewer comments.

| **Item** | **Content** | **Addresses** |
| --- | --- | --- |
| S1 Figure (NEW) | TAT-QRS correlation scatter plot stratified by condition (controls n=17, HF n=16) | R3-Maj-3, R1-Maj-3 |
| S1 Table (NEW) | Geometric characteristics stratified by sex and heart failure status | R1-Maj-5 |
| S2 Figure (NEW) | Correlation matrix: geometric variables vs simulation outputs | R1-Maj-5 |
| S2 Table (NEW) | CGAL meshing parameters (facet_size, cell_size, facet_distance) | R2-2, R2-5 |
| S3 Table | Complete 37-label mesh specification (pre-existing reference in manuscript) | E3 |
| S1 Text | Mechanical simulation boundary conditions (pre-existing reference in manuscript) | E3 |
| S3 Figure (NEW) | Distribution of mean element quality across the 50-heart cohort. Cohort-wide mean 0.153 ± 0.100; no inverted elements found in any mesh. | R2-3 |
| S1 Data (NEW) | Per-model mesh quality statistics for all 50 hearts (CSV). Mesh ID, element count, min/max/mean/SD of tet_qmetric_volume, inverted and near-degenerate element counts. | R2-3 |
| S2 Data | Summary statistics (mean ± SD) for geometric characteristics and simulation outputs, stratified by sex and heart failure status. | E3 |
| S3 Data | Complete two-way ANOVA results for all simulation outputs and geometric metrics, including F-statistics, p-values, and interaction terms. | E3 |
| S4 Data | All pairwise post-hoc comparison results (FDR-corrected). Includes Cohen’s d effect sizes and 95% confidence intervals for all metrics. | E3 |
| S5 Data | Descriptive statistics (mean ± SD) for geometric variables stratified by group (Male/Female × Control/HF). | R1-Maj-5, E3 |
| S6 Data | Spearman correlation coefficients between all geometric variables and simulation outputs, with p-values and significance levels. | R1-Maj-5, E3 |
