# Supplementary material for: Assessing the importance of sex and disease-specific anatomy in electrophysiology and mechanical simulations with a newly developed public virtual cohort of four-chamber heart models
Source: PLoS Comput Biol. 2026 Jun 2;22(6):e1014325. doi: 10.1371/journal.pcbi.1014325 (PMC13252842; doi:10.1371/journal.pcbi.1014325)
Supplement: Peer review history

# Peer review: Decision Letter 0

**DOI:** 10.1371/journal.pcbi.1014325.r001

**Authors:** Daniela Valdez-Jasso

12 Feb 2026

PCOMPBIOL-D-25-02375

Assessing the Importance of Sex and Disease-Specific Anatomy in Electrophysiology and Mechanical Simulations with a Newly Developed Public Virtual Cohort of Four-Chamber Heart Models

PLOS Computational Biology

Dear Dr. Solis-Lemus,

Thank you for submitting your manuscript to PLOS Computational Biology. After careful consideration, we feel that it has merit but does not fully meet PLOS Computational Biology's publication criteria as it currently stands. Therefore, we invite you to submit a revised version of the manuscript that addresses the points raised during the review process.

Please submit your revised manuscript by Apr 14 2026 11:59PM. If you will need more time than this to complete your revisions, please reply to this message or contact the journal office at ploscompbiol@plos.org. When you're ready to submit your revision, log on to https://www.editorialmanager.com/pcompbiol/ and select the 'Submissions Needing Revision' folder to locate your manuscript file.

Please include the following items when submitting your revised manuscript:

* A letter that responds to each point raised by the editor and reviewer(s). You should upload this letter as a separate file labeled 'Response to Reviewers'. This file does not need to include responses to formatting updates and technical items listed in the 'Journal Requirements' section below.

* A marked-up copy of your manuscript that highlights changes made to the original version. You should upload this as a separate file labeled 'Revised Manuscript with Track Changes'.

* An unmarked version of your revised paper without tracked changes. You should upload this as a separate file labeled 'Manuscript'.

If you would like to make changes to your financial disclosure, competing interests statement, or data availability statement, please make these updates within the submission form at the time of resubmission. Guidelines for resubmitting your figure files are available below the reviewer comments at the end of this letter

We look forward to receiving your revised manuscript.

Kind regards,

Daniela Valdez-Jasso, Ph.D.

Academic Editor

PLOS Computational Biology

Dimitrios Vavylonis

Section Editor

PLOS Computational Biology

**Additional Editor Comments:**

The reviewers acknowledged the relevance of the study and expressed interest in the manuscript; however, reviewers raised concerns regarding the lack of validation and aspects of the statistical analysis, which should be addressed in a revised version.

**Journal Requirements:**

If the reviewer comments include a recommendation to cite specific previously published works, please review and evaluate these publications to determine whether they are relevant and should be cited. There is no requirement to cite these works unless the editor has indicated otherwise.

1) Please ensure that the CRediT author contributions listed for every co-author are completed accurately and in full.

At this stage, the following Authors/Authors require contributions: Jose Alonso Solis-Lemus, Cristoph Augustin, Rosie K. Barrows, Cesare Corrado, Nishant Lahoti, Natalie Montarello, Steven A. Niederer, Pras Pathmanathan, Gernot Plank, Abdul Qayyum, Shahrohk Rahmani, Ronak Rajani, Cristobal Rodero, Caroline Roney, Marina Strocchi, Hao Xu, and Alistair Young. Please ensure that the full contributions of each author are acknowledged in the "Add/Edit/Remove Authors" section of our submission form.

The list of CRediT author contributions may be found here: https://journals.plos.org/ploscompbiol/s/authorship#loc-author-contributions

2) We ask that a manuscript source file is provided at Revision. Please upload your manuscript file as a .doc, .docx, .rtf or .tex. If you are providing a .tex file, please upload it under the item type u2018LaTeX Source Fileu2019 and leave your .pdf version as the item type u2018Manuscriptu2019.

3) We have noticed that you have uploaded Supporting Information files, but you have not included a list of legends. Please add a full list of legends for your Supporting Information files after the references list.

4) Some material included in your submission may be copyrighted. According to PLOSu2019s copyright policy, authors who use figures or other material (e.g., graphics, clipart, maps) from another author or copyright holder must demonstrate or obtain permission to publish this material under the Creative Commons Attribution 4.0 International (CC BY 4.0) License used by PLOS journals. Please closely review the details of PLOSu2019s copyright requirements here: PLOS Licenses and Copyright. If you need to request permissions from a copyright holder, you may use PLOS's Copyright Content Permission form.

Please respond directly to this email and provide any known details concerning your material's license terms and permissions required for reuse, even if you have not yet obtained copyright permissions or are unsure of your material's copyright compatibility. Once you have responded and addressed all other outstanding technical requirements, you may resubmit your manuscript within Editorial Manager.

Potential Copyright Issues:

i) Figure 1. Please confirm whether you drew the images / clip-art within the figure panels by hand. If you did not draw the images, please provide (a) a link to the source of the images or icons and their license / terms of use; or (b) written permission from the copyright holder to publish the images or icons under our CC BY 4.0 license. Alternatively, you may replace the images with open source alternatives. See these open source resources you may use to replace images / clip-art:

- https://commons.wikimedia.org

- https://openclipart.org/.

5) In the online submission form, you indicated that The resulting virtual cohort captures key anatomical variability and is publicly available at https://doi.org/10.5281/zenodo.17048090. All PLOS journals now require all data underlying the findings described in their manuscript to be freely available to other researchers, either

1. In a public repository

2. Within the manuscript itself

3. Uploaded as supplementary information.

This policy applies to all data except where public deposition would breach compliance with the protocol approved by your research ethics board. If your data cannot be made publicly available for ethical or legal reasons (e.g., public availability would compromise patient privacy), please explain your reasons by return email and your exemption request will be escalated to the editor for approval. Your exemption request will be handled independently and will not hold up the peer review process, but will need to be resolved should your manuscript be accepted for publication. One of the Editorial team will then be in touch if there are any issues.

6) Please amend your detailed Financial Disclosure statement. This is published with the article. It must therefore be completed in full sentences and contain the exact wording you wish to be published.

1) State what role the funders took in the study. If the funders had no role in your study, please state: "The funders had no role in study design, data collection and analysis, decision to publish, or preparation of the manuscript."

2) If any authors received a salary from any of your funders, please state which authors and which funders..

If you did not receive any funding for this study, please simply state: u201cThe authors received no specific funding for this work.u201d

7)  Please ensure that the files are uploaded in the online submission form in a correct numerical order.

**Reviewers' comments:**

Reviewer's Responses to Questions

**Comments to the Authors:**

**Please note here if the review is uploaded as an attachment.**

Reviewer #1: Please see the attached comments.

Reviewer #2: review is also attached.

The paper presents a cohort of patient-specific virtual models of human hearts suitable for numerical simulations. Starting from medical images, the geometry of the heart is reconstructed via a multi-step process, in which the data are segmented and labeled, and successively, the mesh is created by interfacing with a third-party software (CGAL). The produced dataset is balanced in terms of sex and includes patients with heart disease.

A few clarifications can improve the quality of the manuscript:

1) Are the reconstructed meshes referred to the same timepoint of the cardiac cycle? What is the internal pressure at that instant? Otherwise, how are the models aligned to perform and compare the simulations?

2) Which type of finite elements are supported by the mesh-generator?

3) Have the authors checked the quality of the generated meshes, for instance, by computing the determinant of the Jacobian of the geometrical map? (or the element distortion)

4) Have the authors assessed the quality of the reconstructed geometry compared to the clinical images?

5) Do the users have the possibility of controlling and adapting the mesh by varying the density in specific (user-defined) regions?

Several references are not correctly formatted with respect to the page size; I guess this will be solved during the production stage.

Reviewer #3: The manuscript by Solis-Lemus, Barrows, and co-workers outlines a methodology to generate and anatomical models of patient-specific hearts to assess sex-based differences in healthy and failing hearts. The paper presents results that there are sex-related differences in the mechical and electrophysiological responses to simulation that arises from the anatomical differences alone. This work contributes to the current need in personalized medicine for cardiovascular diseases. However, there are significant issues with the manuscript that need to be addressed prior to publication:

Major Issues

1) The last two paragraph of the introduction section are too long. I would say the very last paragraph is not needed. The second to last paragraph has too much methodology for an intro. For example, sample size numbers don't really belong here. Also, these two paragraphs jump tenses too much.

2) The main issue with the paper is lack of proper validation. There is comparison of lung volumes and ephys between groups, but there is no validation back to patient data. While in the future, the idea of in silico clinical trials is very important, this paper seems to exhibit most of the weaknesses that are the barrier to entry of this technology.

3) There should be a way to get at an estimate for the QRS duration without having to model the full torso. This is really needed to allow the work to be validated against physiological data.

4) Fig 5 is not informative. All I can see are columns of images that to my eye look completely identical. If the reader is supposed to see differences they need to be properly blown up and pointed out. Alternatively, move this to the supplemental if it is not essential.

5) Looking at the data in Fig 6, this does not look like a proper validation. There are plenty of blue (control) dots outside the gray band. And plenty or red dots inside the gray band. If this is to be taken as validation it is very qualitative. Maybe providing distributions from MRI studies?

6) Section 5.3 - given the point above, this was not a convincing argument.

7) Line 233 states that the UK Biobank defines the reference ventricle volumes. It is unclear whether this

definition that was based on only Caucasians is an appropriate standard for the patient demographics in

this study. The paper provided by the link is only Caucasians which seems to be problematic given the stated scope of this paper.

Minor Issues

- In figure 7b, it is quite difficult to distinguish which line of comparison corresponds to which parameter.

- Lines 350-352, is this referencing figure 7a? It is unclear whether "HF females showed the greatest

relative differences" is in comparison to HF males or to control females.

- The acronym CM&S is not necessary and makes it harder to read the paper. Just use the words.

- citation numbers are used as nouns, which makes reading the paper awkward. This would work if the citation style included author names, but reading "[13] did this" is very strange.

- line 261 HF has strange spacing

While the paper addresses an important aspect of furthering the techniques to create in silico clinical trials, there are major issues with validation that follows the same pattern as the barriers in this field.

**********

**Have the authors made all data and (if applicable) computational code underlying the findings in their manuscript fully available?**

The PLOS Data policy requires authors to make all data and code underlying the findings described in their manuscript fully available without restriction, with rare exception (please refer to the Data Availability Statement in the manuscript PDF file). The data and code should be provided as part of the manuscript or its supporting information, or deposited to a public repository. For example, in addition to summary statistics, the data points behind means, medians and variance measures should be available. If there are restrictions on publicly sharing data or code —e.g. participant privacy or use of data from a third party—those must be specified.

Reviewer #1: Yes

Reviewer #2: Yes

Reviewer #3: Yes

**********

PLOS authors have the option to publish the peer review history of their article (what does this mean?). If published, this will include your full peer review and any attached files.

If you choose “no”, your identity will remain anonymous but your review may still be made public.

**Do you want your identity to be public for this peer review?** For information about this choice, including consent withdrawal, please see our Privacy Policy.

Reviewer #1: No

Reviewer #2: No

Reviewer #3: No

[NOTE: If reviewer comments were submitted as an attachment file, they will be attached to this email and accessible via the submission site. Please log into your account, locate the manuscript record, and check for the action link "View Attachments". If this link does not appear, there are no attachment files.]

**Figure resubmission:**

While revising your submission, we strongly recommend that you use PLOS’s NAAS tool (https://ngplosjournals.pagemajik.ai/artanalysis) to test your figure files. NAAS can convert your figure files to the TIFF file type and meet basic requirements (such as print size, resolution), or provide you with a report on issues that do not meet our requirements and that NAAS cannot fix.

After uploading your figures to PLOS’s NAAS tool - https://ngplosjournals.pagemajik.ai/artanalysis, NAAS will process the files provided and display the results in the "Uploaded Files" section of the page as the processing is complete. If the uploaded figures meet our requirements (or NAAS is able to fix the files to meet our requirements), the figure will be marked as "fixed" above. If NAAS is unable to fix the files, a red "failed" label will appear above. When NAAS has confirmed that the figure files meet our requirements, please download the file via the download option, and include these NAAS processed figure files when submitting your revised manuscript.

**Reproducibility:**

To enhance the reproducibility of your results, we recommend that authors of applicable studies deposit laboratory protocols in protocols.io, where a protocol can be assigned its own identifier (DOI) such that it can be cited independently in the future. Additionally, PLOS ONE offers an option to publish peer-reviewed clinical study protocols. Read more information on sharing protocols at https://plos.org/protocols?utm_medium=editorial-email&utm_source=authorletters&utm_campaign=protocols

Attachment Submitted filename: renamed_98ef9.docx

Attachment Submitted filename: PCOMPBIOL-D-25-02375_rev.pdf

# Author comment: Author response to Decision Letter 1

**DOI:** 10.1371/journal.pcbi.1014325.r002

31 Mar 2026

Attachment Submitted filename: response_to_reviewers_update.docx

# Peer review: Decision Letter 1

**DOI:** 10.1371/journal.pcbi.1014325.r003

**Authors:** Daniela Valdez-Jasso

11 May 2026

Dear Dr Solis-Lemus,

We are pleased to inform you that your manuscript 'Assessing the Importance of Sex and Disease-Specific Anatomy in Electrophysiology and Mechanical Simulations with a Newly Developed Public Virtual Cohort of Four-Chamber Heart Models' has been provisionally accepted for publication in PLOS Computational Biology.

Before your manuscript can be formally accepted you will need to complete some formatting changes, which you will receive in a follow up email. A member of our team will be in touch with a set of requests. In addition, please consider following the recommendation of Reviewer 3, who has suggested moving Figure 5 to Supplemental Data.

Please note that your manuscript will not be scheduled for publication until you have made the required changes, so a swift response is appreciated.

IMPORTANT: The editorial review process is now complete. PLOS will only permit corrections to spelling, formatting or significant scientific errors from this point onwards. Requests for major changes, or any which affect the scientific understanding of your work, will cause delays to the publication date of your manuscript.

Should you, your institution's press office or the journal office choose to press release your paper, you will automatically be opted out of early publication. We ask that you notify us now if you or your institution is planning to press release the article. All press must be co-ordinated with PLOS.

Thank you again for supporting Open Access publishing; we are looking forward to publishing your work in PLOS Computational Biology.

Best regards,

Daniela Valdez-Jasso, Ph.D.

Academic Editor

PLOS Computational Biology

Dimitrios Vavylonis

Section Editor

PLOS Computational Biology

***********************************************************

Reviewer's Responses to Questions

**Comments to the Authors:**

**Please note here if the review is uploaded as an attachment.**

Reviewer #2: nothing to add

Reviewer #3: I still feel that there is no need for Fig. 5 in the main body of the paper, but otherwise the authors have addressed all of my concerns.

**********

**Have the authors made all data and (if applicable) computational code underlying the findings in their manuscript fully available?**

The PLOS Data policy requires authors to make all data and code underlying the findings described in their manuscript fully available without restriction, with rare exception (please refer to the Data Availability Statement in the manuscript PDF file). The data and code should be provided as part of the manuscript or its supporting information, or deposited to a public repository. For example, in addition to summary statistics, the data points behind means, medians and variance measures should be available. If there are restrictions on publicly sharing data or code —e.g. participant privacy or use of data from a third party—those must be specified.

Reviewer #2: Yes

Reviewer #3: None

**********

PLOS authors have the option to publish the peer review history of their article (what does this mean?). If published, this will include your full peer review and any attached files.

If you choose “no”, your identity will remain anonymous but your review may still be made public.

**Do you want your identity to be public for this peer review?** For information about this choice, including consent withdrawal, please see our Privacy Policy.

Reviewer #2: No

Reviewer #3: No

# Editor report: Acceptance letter

**DOI:** 10.1371/journal.pcbi.1014325.r004

**Authors:** Daniela Valdez-Jasso

PCOMPBIOL-D-25-02375R1

Assessing the Importance of Sex and Disease-Specific Anatomy in Electrophysiology and Mechanical Simulations with a Newly Developed Public Virtual Cohort of Four-Chamber Heart Models

Dear Dr Solís-Lemus,

I am pleased to inform you that your manuscript has been formally accepted for publication in PLOS Computational Biology. Your manuscript is now with our production department and you will be notified of the publication date in due course.

The corresponding author will soon be receiving a typeset proof for review, to ensure errors have not been introduced during production. Please review the PDF proof of your manuscript carefully, as this is the last chance to correct any errors. Please note that major changes, or those which affect the scientific understanding of the work, will likely cause delays to the publication date of your manuscript.

Soon after your final files are uploaded, unless you have opted out, the early version of your manuscript will be published online. The date of the early version will be your article's publication date. The final article will be published to the same URL, and all versions of the paper will be accessible to readers.

For Research, Software, and Methods articles, you will receive an invoice from PLOS for your publication fee after your manuscript has reached the completed accept phase. If you receive an email requesting payment before acceptance or for any other service, this may be a phishing scheme. Learn how to identify phishing emails and protect your accounts at https://explore.plos.org/phishing.

Thank you again for supporting PLOS Computational Biology and open-access publishing. We are looking forward to publishing your work!

With kind regards,

Anita Estes

PLOS Computational Biology | Carlyle House, Carlyle Road, Cambridge CB4 3DN | United Kingdom ploscompbiol@plos.org | Phone +44 (0) 1223-442824 | ploscompbiol.org | @PLOSCompBiol
